# ABP-B9, a new strain of *Pseudomonas seleniipraecipitans* with biostimulant activity

**DOI:** 10.3389/fpls.2025.1561298

**Published:** 2025-06-25

**Authors:** Agustina Bernal-Vicente, Pedro Joaquín Sánchez-Pujante, Pedro Diaz-Vivancos, Livia Donaire, Miguel A. Aranda, Yolanda Hernando

**Affiliations:** ^1^ R&D Department, Abiopep S.L., Murcia, Spain; ^2^ Plant Breeding Department, Centro de Edafología y Biología Aplicada del Segura (CEBAS-CSIC), Murcia, Spain; ^3^ Stress Biology and Plant Pathology Department, Centro de Edafología y Biología Aplicada del Segura (CEBAS-CSIC), Murcia, Spain

**Keywords:** biostimulation, celery, leafy vegetables, lettuce, PGPR, spinach

## Abstract

**Introduction:**

Microorganisms are emerging as key agents in sustainable agriculture due to their ability to enhance crop productivity while reducing environmental impact. Among them, *Pseudomonas* spp. are well known for promoting plant growth through mechanisms such as phytohormone production and improved nutrient availability. This study describes the characterization of the strain ABP-B9, isolated from the rhizosphere of commercial lettuce crops.

**Materials and methods:**

ABP-B9 was evaluated under both field and controlled conditions to assess its plant growth-promoting effects. Parameters such as root development, photosynthetic efficiency, flavonoid content, nitrogen status, and the production of indole-3-acetic acid (IAA) and siderophores were measured. Whole-genome sequencing and phylogenetic analysis were also performed.

**Results:**

Field trials showed that ABP-B9 enhanced crop yield in lettuce, spinach, and celery, improving root development, photosynthetic efficiency, flavonoid levels, and nitrogen status. The production of IAA and siderophores was confirmed *in vitro*. Plant responses were observed as early as five days after application. Genomic analysis revealed that ABP-B9 belongs to the *Pseudomonas* genus and is closely related to *P. seleniipraecipitans*. Its genome (4,602,210 bp; 61.46% GC content) includes 4,247 protein-coding genes, 12 rRNAs, and 66 tRNAs.

**Discussion:**

ABP-B9 is a novel, non-pathogenic *Pseudomonas* strain with clear biostimulant activity. Its ability to enhance plant growth and increase crop yield, combined with its safety profile, supports its potential use in sustainable agriculture. Future studies should explore its application across different crops and environmental conditions.

## 1 Introduction

In a deeply needed context of sustainable alternatives for increasing agricultural productivity and reducing environmental impact, microorganisms have emerged as promising allies. Among them, bacteria of the genus *Pseudomonas* stand out for their versatility and capacity to play fundamental roles in promoting plant growth. The genus *Pseudomonas* comprises more than 320 species (Parte et al., 2020), divided into nine major groups based on Multilocus Sequence Analyses (MLSA). These bacteria are Gram-negative rods, straight or slightly curved, and saprophytic. They can be found in multiple ecosystems (aquatic or terrestrial) due to their ability to metabolize a wide range of substrates, allowing them to utilize different compounds as carbon and energy sources (Higuera et al., 2023). They do not form spores, and their optimal growth temperature range from 25 to 30°C, although they can grow from 5 to 42°C. They require a neutral pH and do not grow under acidic conditions (pH ≤ 4.5). Their polar flagella enable active movement in liquid media.

The genus *Pseudomonas* encompasses a wide variety of bacterial species known for their adaptability to diverse environments and diversified metabolic capacity, several of which have been the subject of intense research due to their ability to colonize plant roots and establish beneficial symbiotic relationships, interacting associatively with plants. It is believed that these bacteria are attracted and stimulated by the presence and composition of different root exudates (Drogue et al., 2012). Some members of this genus promote growth and can activate systemic induced resistance in plants (SIR) (Schwanemann et al., 2020). In this sense, their potential as agricultural biostimulants has sparked growing interest in the scientific and agricultural research communities. Thus, numerous *Pseudomonas* species have been included in the group of Plant Growth-Promoting Rhizobacteria (PGPR) due to their biostimulant activity, which can be exerted directly by facilitating plant access to compounds synthesized by themselves, such as indole acetic acid, which increases root growth, or through biochemical processes, including production of other phytohormones and/or nitrogen fixation (Rodríguez-Blanco et al., 2015), iron chelation, and phosphorus solubilization. Furthermore, they can help plants tolerate abiotic stress due to salinity or the presence of heavy metals, among others stress factors, by using various mechanisms such as antioxidant compounds production (Karnwal, 2021). It should be noted that the growth-promoting activity of PGPR is dependent on agricultural management and soil type (Brock et al., 2013; Schreiter et al., 2014).

Within the genus *Pseudomonas*, *Pseudomonas seleniipraecipitans* has attracted researchers’ attention due to its unique ability to metabolize selenium, a trace element that can be both essential and toxic to living organisms depending on its form and concentration. Potential applications in biotechnology and bioremediation have been attributed to this bacterium due to its ability to precipitate selenium, converting it into a less toxic form, elemental selenium, which is more easily assimilated by other organisms (Hunter, 2014), thus contributing to the mitigation of selenium toxicity in aquatic and terrestrial ecosystems. However, to date, no evidence has been found in the scientific literature indicating that *Pseudomonas seleniipraecipitans* has biostimulant effects on plants. Despite the interest in *Pseudomonas seleniipraecipitans* for its ability to reduce selenite and selenate, the scientific information available on this species is very limited. To date, no studies have been found that thoroughly detail its mechanisms of action, applications in bioremediation, or possible biostimulant effects on plants. This study expands the knowledge of this bacterium by evaluating its potential in the agricultural field.

The present study describes the characterization of ABP-B9, a representative of a new strain of *Pseudomonas seleniipraecipitans* isolated from the lettuce rhizosphere in a commercial crop from Aguilas (Murcia, Spain). Lettuce is a very important winter crop in the Murcia region, with Spain being the leading lettuce exporter worldwide (FAOSTAT, 2021). Here we show that ABP-B9 has a biostimulant capacity on lettuce, as well as in spinach and celery crops under commercial agronomic conditions. We determined the complete sequence of the ABP-B9 genome, explored its genetic traits beneficial to plants, and conducted comparative phylogenomic, phenotypic, and chemotaxonomic analyses with related taxa, with all data strongly supporting ABP-B9 as a representative isolate of a new strain of *Pseudomonas seleniipraecipitans* with plant biostimulant capacity.

## 2 Materials and methods

### 2.1 ABP-B9 isolation and characterization

ABP-B9 was isolated from the root of a lettuce plant cultivated in an open field near Águilas (Murcia, Southeast Spain). The root was washed with tap water and sterilized with 10% of sodium hypochlorite for 10 min and then cut into small pieces that were placed in Petri dishes containing Luria-Bertani medium (LB). Petri dishes were incubated at 28°C for three days, and the morphologically distinct bacterial colonies were isolated and characterized by PCR amplification of a region of the 16S RNA encoding gene. The PCR was carried out in a total volume of 20 µL using primers 8F (Turner et al.,1999), 800R (Leis et al., 2019) (**Supplementary Table S1**), and the NXT Taq PCR Kit (EURx Ltd., Poland). The PCR products were analyzed via 0.7% agarose gel electrophoresis, and DNAs of the expected sizes (800 pb) were recovered from the gels using the GeneClean turbo kit (MP Biomedicals, Europe), according to the manufacturer’s recommendations. The sequencing of the PCR products was performed by Sanger sequencing (Stab Vida S.L., Portugal) using the same PCR primers.

Morphological and phenotypic characterizations were performed by the Spanish Type Culture Collection (CECT, University of Valencia, Spain). Cell morphology was analyzed by phase contrast microscopy with a Leica DMRB fluorescence microscope, using wet preparations obtained from an ABP-B9 culture grown in LB for 48 h on both plate and liquid media. The phenotypic characterization was performed using API 20NE strips following the manufacturer’s recommendations (BioMerieux).

The functional characterization of ABP-B9 included the assessment of hydrogen cyanide (HCN) production, phosphate (P) and potassium (K) solubilization, and nitrogen fixation. HCN production was evaluated following Bakker and Schippers (1987) by growing ABP-B9 on King B medium supplemented with glycine, placing a filter paper soaked in picric acid-sodium carbonate on the plate, and incubating it at 28°C for up to 4 days; a colour change from yellow to red indicated a positive result. Phosphate solubilization was tested using Pikovskaya’s agar with calcium phosphate, where the formation of a transparent halo after 4–5 days at 28°C confirmed solubilization (Widawati and Suliasih, 2019). Nitrogen fixation was determined using the Dobereiner method (Boddey and Dobereiner, 1995), by incubating ABP-B9 in nitrogen-free bromothymol blue (NFB) semisolid medium at 28°C for 5–7 days; a foggy ring below the surface and a colour change due to ammonia production indicated positive nitrogen fixation. Potassium solubilization was assessed using modified Pikovskaya’s agar (Granja and Fernanda, 2010), incubating the culture at 28°C for 5 days, where K solubilization was identified by the formation of a yellow halo around the colonies.

### 2.2 *In vitro* plant culture for metabolomic analyses

ABP-B9 was screened for different plant growth-promoting activities. These experiments used *in vitro* grown lettuce (variety Amenas; Rijk Zwaan, Spain), cucumber (variety Wisconsin SMR-58; Mascarell Semillas, Spain), and tomato (variety Caniles; Syngenta, Spain). Plants were grown in culture tubes with filter paper supports in presence of liquid medium (Murashige and Skoog, 2006) including Gamborg vitamins (Gamborg et al., 1968); Sucrose: 30 g/L; Casamino acids: 0.25 g/L; and pH: 5.9-6. The seeds were previously sterilized: lettuce seeds were treated with 20% sodium hypochlorite + 0.1% Tween 20 for 20 minutes and pre-germinated at 18°C for three days; cucumber seeds were treated with a mixture of 20% sodium hypochlorite + 1mM HCl + 0.1% Tween 20 for 20 minutes and pre-germinated at 25°C for three days; tomato seeds were treated with a mixture of 20% sodium hypochlorite + 0.1% Tween 20 for 20 minutes and pre-germinated at 25 °C for three days. Once the seeds had germinated, they were placed on filter paper inside glass test tubes containing 15 mL of liquid medium (described above) and allowed to grow for seven days at 26°C with a photoperiod of 16 hours of light and 8 hours of darkness. After this period, they were inoculated with 2 mL of the corresponding treatment. The B9 treatment consisted of the application of 2 mL of a culture of the ABP-B9 isolate in LB medium at 28°C during 48 hours in darkness at 150 rpm (≥10^7^ CFU/mL). The MEC treatment consisted of the application of 2 mL of ABP-B9-inoculated medium incubated for 48 hours, after which the bacteria were removed by centrifugation in order to evaluate the effect of the metabolites released by the bacteria in its absence. The control treatment consisted of the addition of 2 mL of sterile distilled water. The plants were left to grow for approximately two weeks, and after this period, differences in the development of both the aerial parts and the root systems were evaluated. The treatments included at least 10 plants, with at least three repetitions.

The ability to produce indole-3-acetic acid (IAA) and siderophores was measured in liquid media from lettuce, tomato, and cucumber plants grown and inoculated as described above. The IAA production ability was tested spectrophotometrically (Gordon and Weber, 1951). Briefly, 5 mL of the plant growth media was pelleted by centrifugation for 20 min at 4400 rpm, and 1 mL of the supernatant was assayed for the presence of IAA using 2 mL of Salkowski reagent (1 mL of 0.5 M FeCl_3_ in 50 mL of 35% HClO_4_). After 30 min of incubation in darkness, the absorbance at 530 nm was measured in an Epoch 2 Microplate Spectrophotometer (BioTek Instruments, Vermont, USA). The IAA content was determined using a standard curve of synthetic IAA at different concentrations (Duchefa Biochemie, Haarlem, The Netherlands) ranging from 0 to 50 µg/mL. The ferric chloride (FeCl_3_) test was used for the detection of siderophores. One mL of FeCl_3_ solution (2%) was added to 1 mL of the previously described supernatant, and absorbance was recorded from 420 nm to 500 nm with the Epoch 2 Microplate Spectrophotometer. A peak between 420–480 nm in ferrated siderophores indicates a hydroxamate type of siderophore, while a peak at 495 nm indicates a catecholate type of siderophore.

Metabolomic analyses were performed in roots from plants grown in liquid media under three treatments: presence of ABP-B9 (B9), absence of ABP-B9 (LB), and the presence of ABP-B9-inoculated media for 48h, after which the bacteria was removed by centrifugation (MEC). A set of three samples from each treatment were analyzed by liquid chromatography electrospray ionization (positive ionization mode) quadrupole time-of-flight mass spectrometry (LC-ESI-QTOF-MS/MS) using a Waters ACQUITY UPLC I-Class System (Waters Corp.) coupled to a Bruker Daltonics QToFMS mass spectrometer (maXis impact Series with a resolution ≥55000 FWHM Bruker Daltonics) at the “Fundación Medina Chemistry Services” (Granada, Spain). Correlation and clustering between samples were determined by Partial Least Squares Discriminant Analysis (PLS-DA) and hierarchical clustering heatmap (displaying the top 25 variable mass features) using the software MetaboAnalyst 6.0 (https://www.metaboanalyst.ca). Based on the differentially expressed m/z and its retention times, the “Fundacion Medina Chemistry Services”, using the Dictionary of Natural Products Version 30.2, provided a list of candidate compounds (based on their molecular formula) for each detected peak. Those candidate compounds were then analyzed in the annotated features module of MetaboAnalyst 6.0 in order to identify metabolic pathways differentially affected using the Mummichog algorithm, which provides direct mapping using existing pathway databases (Li et al., 2013). The following criteria were used: molecular weight tolerance set at 5 ppm, primary ions enforced, and *Arabidopsis thaliana* as the pathway library (Jurado-Mañogil et al., 2023).

### 2.3 Field assays

Field assays were carried out with three different crop species (lettuce, celery and spinach). The aim of these trials was to analyze the effect of the application of ABP-B9 on the quality and performance of the aforementioned crops. Trials were designed for each crop including control (untreated plants) and ABP-B9 treated plants. Each treatment included 120 plants in the case of lettuce and celery crops, and 2100 plants in the case of spinach. The experimental design consisted of three replicates of 40 plants each in the case of lettuce and celery, and 700 plants per replicate for spinach. Lettuce cultivation was carried out at the “Garrobillo” farm, located in Águilas (Murcia, Spain), from November to February 2022-23. Celery cultivation was conducted at the “Grima” farm (plot named P5.1), located in Águilas, from October to February 2021-2022, whereas spinach cultivation was carried out at the “Inicio” farm (plot named P 13), located in Fuente Álamo (Murcia, Spain), from January to February 2022. The lettuce trial was performed using the commercial iceberg variety Amenas (Rijk Zwaan, Spain). The celery variety used was PH 535 (Ramiro Arnedo, Spain) and the spinach variety El Giga (Syngenta, Spain). Lettuce and celery seedlings were grown under commercial conditions until they reached the appropriate size for transplanting into the field, 37 days for lettuce and 56 days for celery. The trays used were made of polystyrene with 294 cells, a surface area of 46×71 cm, and a cell volume of 22 cm³. In the case of celery cultivation, the trays had the same surface area and cell volume but a higher plant density, as these trays contained 345 cells. The type of substrate used in both cases was Kekkila MSM 010 WL1 RA 323 peat. The spinach was directly sown in the field with a planting density of 7.5 million plants/ha. The planting density of lettuce was 86,000 plants/ha, and in the case of celery, 94,444 plants/ha. The different treatments were distributed in a completely randomized block design and the treatments were separated from each other with untreated plants to avoid possible contaminations. The lettuce and celery plants were treated with bacteria at the seedling stage five days before transplanting to the field. The treatment consisted of direct application of 1.7 mL/seedling of a bacterial culture, grown in LB medium at 28°C during 48 hours in darkness and diluted in sterile distilled water to a concentration of ≥10^7^ CFU/mL, applied to the root ball. The control treatment received 1.7 mL of sterile distilled water. In the case of the spinach crop, the application of the treatments was carried out with a 10 L capacity backpack sprayer when the spinach seedlings had developed the first true leaf (20 days after sowing). Each plant, as for the other crops, received approximately 1.7 mL of solution.

At the time of transplanting lettuce seedlings to the field, the aerial and root system development of 10 seedlings from each treatment was analysed. After harvest—at 75 days for lettuce, 100 days for celery, and 90 days for spinach—the effect of the treatments on crop development was assessed by measuring the weight of the aerial parts, plant height, and dry weight of the root system. For spinach cultivation, 40 whole plants were individually harvested from each replicate, totalling 120 plants per treatment, to evaluate the development of the aerial parts and root system.

After completing the lettuce trial, ten lettuce plants per treatment were harvested and processed following standard storage procedures in a commercial chamber for 16 days. To evaluate the shelf life of lettuce stored under refrigerated conditions (4 ± 1°C, 90–95% RH), quality assessments were conducted at regular intervals (0, 3, 6, 9, 12, and 16 days), which included measurements of weight loss and visual quality based on a rot scale established in our laboratory (**Supplementary Figure S4**). The weight loss of samples was determined by weighing ten heads of lettuce from each treatment at each sampling time, applying the following equation: Weight loss (%) = (W0 − Wt)/W0 × 100, where W0 is the initial sample weight (at day 0) and Wt is the sample weight at each sampling time (Martínez-Sánchez et al., 2024). Statistical analysis was performed at a 95% confidence level (p < 0.05) using one-way ANOVA and Tukey HSD with Statgraphics Plus 5.1 data analysis software. Data are expressed as the mean value of ten samples.

Weight measurements and decay assessments were conducted every three days to evaluate the effect of ABP-B9 application on lettuce shelf life.

To evaluate the presence of ABP-B9 in the root system of treated plants, this microorganism was quantified using qPCR analysis. Specific primers for ABP-B9 (AB-858_F/AB-859_R) were used (**Supplementary Table S1**). qPCR reactions were performed in a StepOnePlus Real-Time PCR System (Applied Biosystems, USA) with FastGene IC Green 2x qPCR Universal Mix (Nippon Genetics, Germany). PCR conditions included denaturation at 95°C (2 min), followed by 40 cycles at specific annealing temperatures at 60°C. Dissociation curves ensured specificity. Copy number, linear regression, and melting curve analysis were conducted using StepOnePlus v2.3 software. The Nadh4 (Navarro et al., 2004) gene from lettuce served as housekeeping control. Statistical differences were analyzed using one-way or two-way ANOVA with Statgraphics Plus 5.1.

In addition, the effect of ABP-B9 on photosynthesis performance in lettuce plants was evaluated in an independent experiment carried out in open field crops in 2021 in Águilas (Murcia, Spain). The trials included both control (untreated plants) and ABP-B9-treated plants. Each treatment consisted of 120 plants of the variety Amenas (Rijk Zwaan, Spain), divided into three replicates. As in the previous experiment, seedlings were grown under commercial conditions until they reached the appropriate size for transplanting into the field. The planting density was 86,000 plants/ha. The different treatments were arranged in a completely randomized block design, with untreated plants used as buffers to avoid possible contamination between treatments. The plants were treated in the same manner as in the previous experiment. At the end of the growing season, leaf samples were taken from 10 plants from each treatment group. The content of chlorophylls, polyphenols (flavonols and anthocyanins), and the nitrogen balance index (chlorophyll/flavonol ratio) were determined using the Dualex Scientifc portable leaf-clip optical sensor (FORCE A; France). In addition, chlorophyll fluorescence parameters were analyzed using a modulated fluorescence fluorimeter (FMS2, Hansatech Instruments, UK), and data on the quantum efficiency of PSII [Y(PSII)], the photochemical quenching coefficient (qP), the non-photochemical quenching coefficient (qNP), and the electron transport rate (ETR) were recorded (Jurado et al., 2024).

One-Way or two-way ANOVA and the Student’s t-test were performed on each field trial to specifically evaluate the impact of ABP-B9 application in the different crops. The data analysis was performed using Statgraphics Plus 5.1. data analysis software. The significance level was set to p < 0.05.

### 2.4 ABP-B9 DNA preparation

ABP-B9 was cultured aerobically in LB medium, with shaking at 28°C. Genomic DNA was extracted using the CTAB method (Doyle, 1991). DNA concentration was determined by a Nanodrop spectrophotometer (Thermo Fisher Scientific, USA) and its quality checked with agarose gel electrophoresis.

### 2.5 Genome sequencing and draft assembly

Eight µg of ABP-B9 DNA was used for the whole-genome sequencing. Sequencing was performed by Macrogen Inc. using PacBio single molecule real-time (SMRT) technology. The sequencing reads were assembled using the RS Hierarchical Genome Assembly Process 3.0 (HGAP) within the SMRT Portal 2.3 (Chin et al., 2013).

### 2.6 Genome annotation

After the draft genome was assembled, the annotation of the genome, including the location of protein-coding sequences, tRNA genes, and rRNA genes, was performed using the National Center for Biotechnology Information (NCBI)’s Prokaryotic Genome Annotation Pipeline 2.0 (PGAP) (Tatusova et al., 2016). The Prokka software tool (v1.12b) (Seemann, 2014) was used to further annotate protein-coding genes. Orthology and functional annotations of proteins were inferred using the evolutionary gene genealogy Non-supervised Orthologous Groups (eggNOG) database (Huerta-Cepas et al., 2019) and the InterPro database (https://www.ebi.ac.uk/interpro/about/interpro/).

### 2.7 Whole-genome comparisons for species identification

The housekeeping genes proposed for the genus *Pseudomonas* for species delimitation are 16S rRNA, gyrB, rpoD and rpoB (Lalucat et al., 2006). A BLASTn search of these genes in ABP-B9 against the NCBI database was carried out to identify to which species each gene showed the highest similarity. The best hit and the next top five hits were selected for whole-genome comparisons. Comparisons were performed using in silico genome analysis methods for bacterial identification that are based on alignment-dependent (Schreiter et al., 2014) and independent approaches (Coutinho et al., 2015). In both approaches, if a value of a metric is above an established threshold, the compared strains are considered to belong to the same taxon. In the present study we calculated the following parameters in order to discriminate *Pseudomonas* from other genera: the Average Nucleotide Identity, based on MUMmer (ANIm) or BLAST (ANIb), the correlation of the tetranucleotide signatures (TETRA), the Orthologous Average Nucleotide Identity (OrthoANI), the digital DNA-DNA hybridization (dDDH), the Average Amino Acid Identity (AAI), and the percentage of GC. ANIb, ANIm and TETRA were calculated using the JSpecies software tool (http://www.imedea.uib.es/jspecies). The recommended species cut-off was 95% for the ANIb and ANIm indices, and higher than 0.99 for the TETRA signature (Richter et al., 2016). OrthoANI was calculated using the ANICalculator tool (https://www.ezbiocloud.net/tools/ani) (Yoon et al., 2017). The recommended cut-off for species demarcation is 95-96%. dDDH and GC content from the sequenced fragments of the different genes were calculated using the web service http://ggdc.dsmz.de (Meier-Kolthoff et al., 2013). For the dDDH parameter, the recommended species threshold is ≥ 70%. AAI was calculated using the web service AAI-profiler (http://ekhidna2.biocenter.helsinki.fi/AAI/) (Medlar et al., 2018). The AAI cut-off point for species demarcation is 95–96% (Konstantinidis and Tiedje, 2005). The bioinformatics resources and tools used in this section are shown in the **Supplementary Table S2**.

### 2.8 Phylogenetic analyses

The same housekeeping genes used for species delimitation were used to perform phylogenetic analyses to infer the phylogenetic position of ABP-B9 within the genus *Pseudomonas*. A MLSA was performed using nucleotide fragment sequences in the following order: 16S rDNA (55 nt), *gyrB* (625 nt), *rpoD* (489 nt), and *rpoB* (435 nt) (Mulet et al., 2010). These sequences were extracted from the complete ABP-B9 genome, the public NCBI database, and the *Pseudomonas* Genome Database (https://www.pseudomonas.com). The Molecular Evolutionary Genetics Analysis Version 7.0 (MEGA7) software (Kumar et al., 2016) was used for the multiple sequence alignment using MUSCLE (Multiple Sequence Comparison by Log-Expectation). The General Time Reversible substitution model plus gamma distributed with invariable sites (GTR+G+I) was selected according to the lowest BIC (Bayesian Information Criterion) score. Phylogeny was generated using the Maximum Likelihood method (Felsenstein, 1981), with 1,000 bootstrap replicates. The final tree was constructed and edited using Interactive Tree of Life (iTOL) v6. The pairwise genetic distances between sequences were calculated using the MEGA7 software. Clustal X (2.1) was used to determine the percent nucleotide identity matrix (Thompson et al., 1997).

### 2.9 Genome mining

The presence of sequences related to integrated plasmids and prophages was studied with the PHASTER tool (Zhou et al., 2011; Arndt et al., 2016). ICEfinder was used to identify mobile genetic elements (Liu et al., 2019). Proksee (Grant et al., 2023), ResFinder (Bortolaia et al., 2020), VFDB (Virulence Factor Database) (Altschul et al., 1997), and VRprofile2 (Wang et al., 2022) were used to identify virulence or antibiotic resistance genes in the ABP-B9 genome. PathogenFinder (http://cge.cbs.dtu.dk/services/PathogenFinder/) was used to identify gene families that correlate with human pathogenicity. The ABP-B9 mycotoxin synthesis ability was evaluated with ToxFinder 1.0. AntiSMASH v6.0 was used for predicting secondary metabolite biosynthetic gene clusters (Blin et al., 2021). Moreover, the RAST annotation server (http://rast.theseed.org/FIG/rast.cgi) was used to predict metabolic pathways annotated in the ABP-B9 genome using the KEGG (Kyoto Encyclopedia of Genes and Genome) as the pathway database. Type VI secretion system components were identified with SecRet6 (Li et al., 2015). The bioinformatics resources and tools used in this section are shown in the **Supplementary Table S2**.

### 2.10 Acute oral toxicity

ABP-B9 acute oral toxicity was tested following the OECD Guidelines to Testing of Chemicals (OECD 423:2001). The test was carried out by the Valencian Institute of Microbiology (www.ivami.com; Bétera, Valencia). The method is based on a stepwise procedure, in which three animals are used at each stage. The absence or presence of mortality in the first stage determines whether the test is concluded or if three animals are dosed with the same, higher, or lower dose. The test was conducted on six female albino mice, which were administered with 1.95 mL/kg of a culture of the ABP-B9 isolate at a concentration of 10^8^ CFU/mL through intraesophageal inoculation.

## 3 Results

### 3.1 Preliminary characterization of ABP-B9

**Figure 1:**
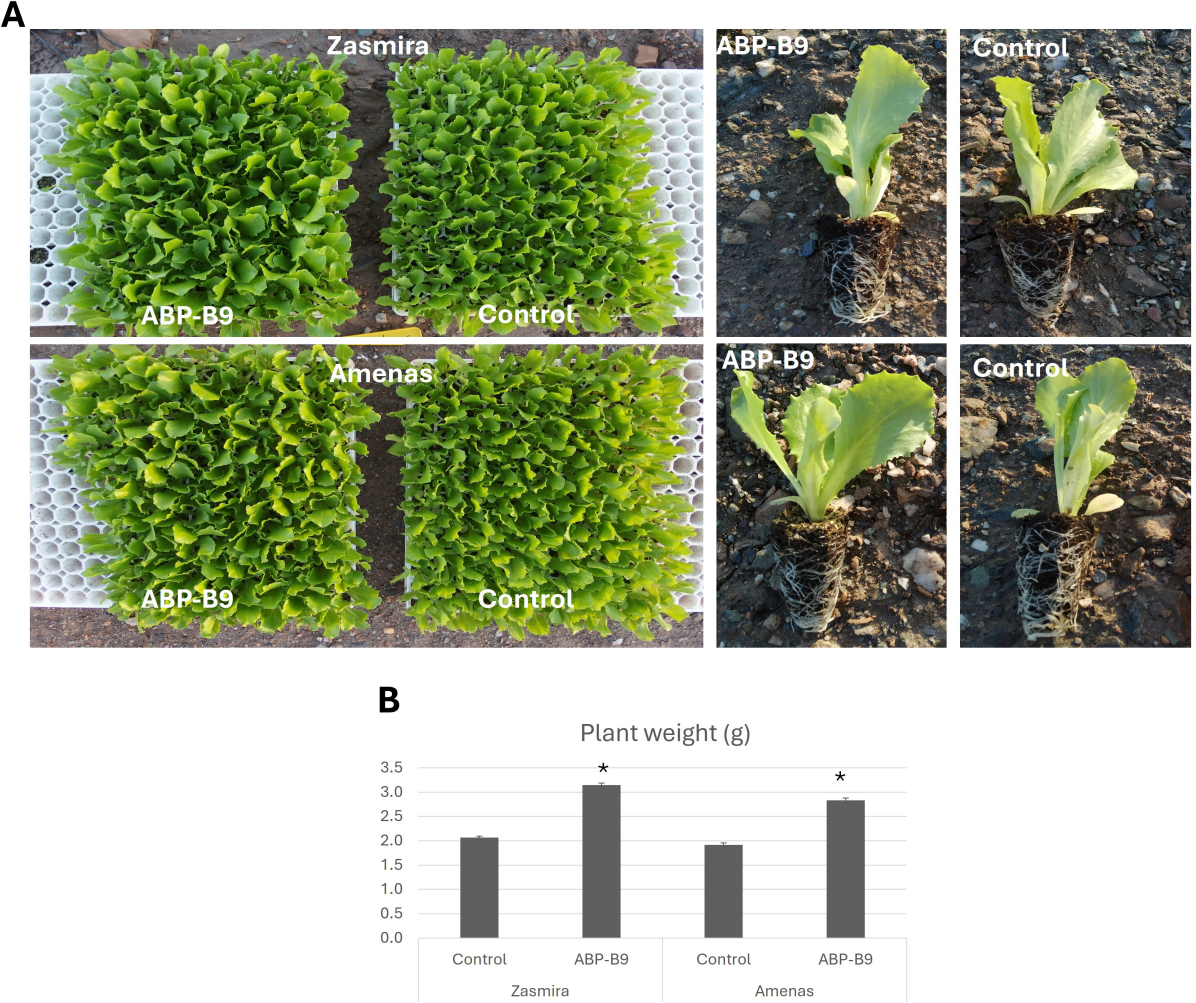
Biostimulant ABP-B9 activity. **(A)** Lettuce plants of the Amenas and Zasmira varieties in the absence (control) and presence of ABP-B9 (ABP-B9) 5 days after applying the treatment. **(B)** Average fresh weight of seedlings of the Zasmira and Amenas varieties five days after the application of ABP-B9. Asterisks indicate significant differences between treatments according to a One-Way ANOVA statistical test (p < 0.05).

**Figure 2:**
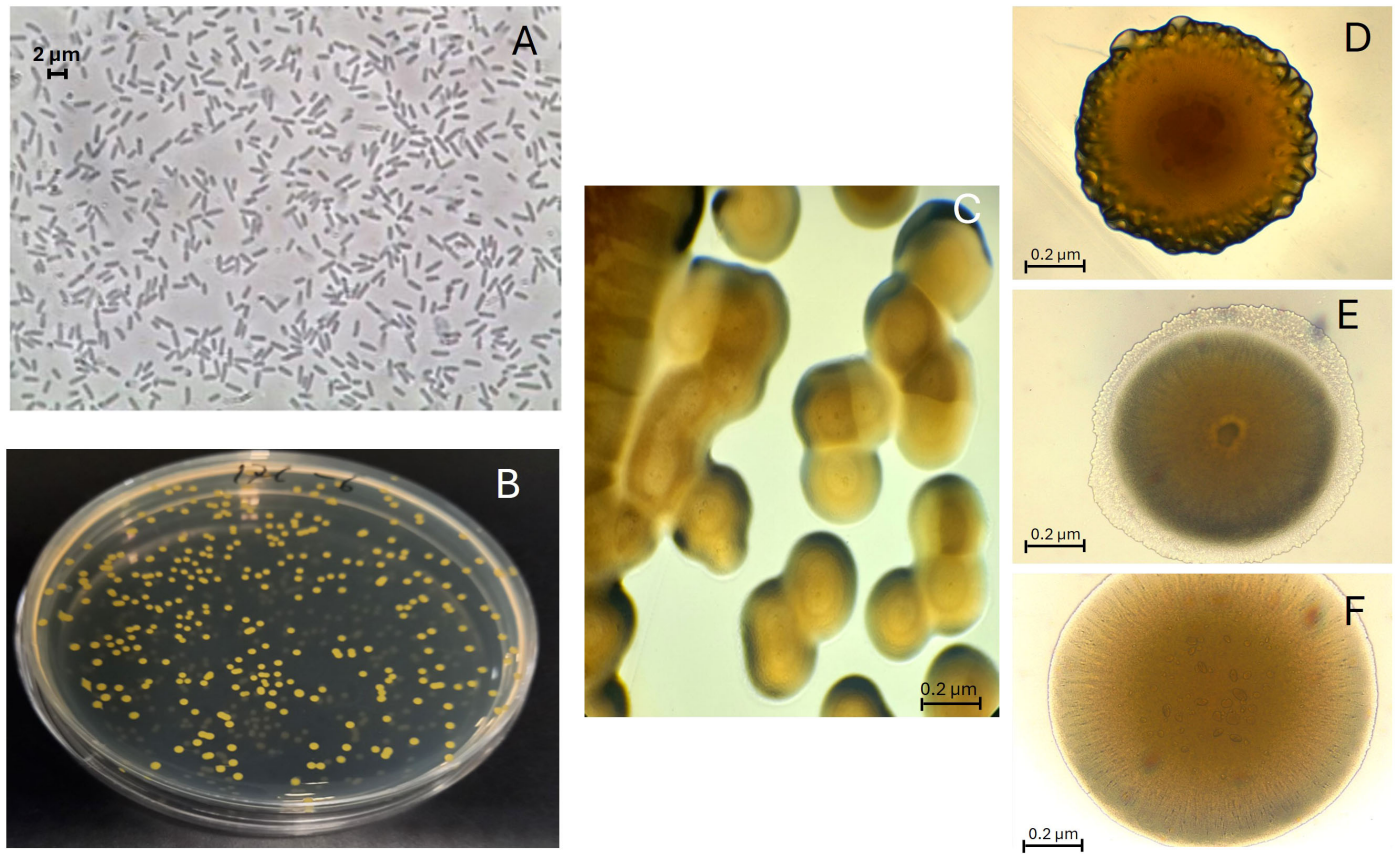
Cellular morphology of ABP-B9. **(A)** Image of phase contrast microscopy of ABP-B9 in LB. A Leica DMRB fluorescence microscope was used. **(B)** Colonies of ABP-B9 on Luria-Bertani (LB) medium plates. **(C)** Colony phase variants of strain ABP-B9 observed under the optical microscope. **(D–F)** Colony morphotype variants.

A field survey was performed in a commercial lettuce crop near Águilas (Murcia, Southeast Spain) to isolate microorganisms present in the root systems of vigorously growing lettuce plants and evaluate their potential as biostimulat agents. The isolate ABP-B9, an endophytic microorganism, showed biostimulant effects on lettuce seedlings (**Figures 1A, B**). Our preliminary study also showed that isolate ABP-B9 shared the basic phenotypic traits of the genus *Pseudomonas:* Gram-negative rod, motility via polar flagella, with a strictly respiratory type of metabolism, and catalase and oxidase activity (**Table 1**). It did not produce water-soluble fluorescent pigments but produced a characteristic water-insoluble yellow pigment on LB medium. Colonies appeared smooth, round, convex, and yellow on LB medium (**Figure 2**). A pure culture of isolate ABP-B9 in LB medium revealed average dimensions of 1.6 ± 0.4 µm in length and 0.5 ± 0.1 µm in width (**Figure 2A**). ABP-B9 showed a colony phase variation in solid medium (**Figures 2B–F**). The colony morphotypes were clearly distinct but had undistinguishable 16S rDNA sequences.

**Table 1:** Classification and general features of isolate ABP-B9.

General features	
Domain	*Bacteria*
Phylum	*Proteobacteria*
Class	*Gammaproteobacteria*
Order	*Pseudomonadales*
Family	*Pseudomonadaceae*
Genus	*Pseudomonas*
Species	*Pseudomonas seleniipraecipitans*
Strain	ABP-B9
Gram Stain	Negative
Cell shape	Rod
Motility	Motile
Spore formation	Non-spore forming
Temperature growth range	4-37°C
Optimal growth temperature	28°C
pH range	4-9
Optimal pH	7
Carbon sources	Monosaccharides, organic acids, alcohols, amino acids, amines
Catalase	+
Oxidase	+
Habitats	Agricultural soils
Salinity	5% (0.86 M)
Oxygen requirement	Aerobic
Biotic relationship	Endophytic
Pathogenicity	Non-pathogenic
Geographical location	Spain
Place of isolation	Águilas
*In vitro* plant growth promoting (PGP) traits	
IAA production	+
Siderophore production	+
Phosphate solubilization	–
Potassium solubilization	–
Fixation of nitrogen	–
HCN production	–

Detected (+)/Not detected (-).

The results of the BioMerieux API 20 NE Gallery System for isolate ABP-B9 were partially consistent with a representative of the species *Pseudomonas fluorescens*, but only with a 92.3% identity (**Supplementary Figure S1**).

### 3.2 ABP-B9 promotes significant metabolic changes and the growth of lettuce roots

Isolate ABP-B9 was screened for different plant growth-promoting activities, including IAA and siderophore production. Additionally, nitrogen fixation, phosphate and potassium solubilization, and hydrogen cyanide (HCN) production, were assessed using specific growth media, showing various plant growth promoting traits (**Table 1**). The analyses conducted under *in vitro* conditions showed that the isolate was unable to fix nitrogen, solubilize phosphorus or potassium, or produce HCN (**Table 1**), which contrasts with results of the genomic data mining described below (**Supplementary Table S5**).

**Figure 3:**
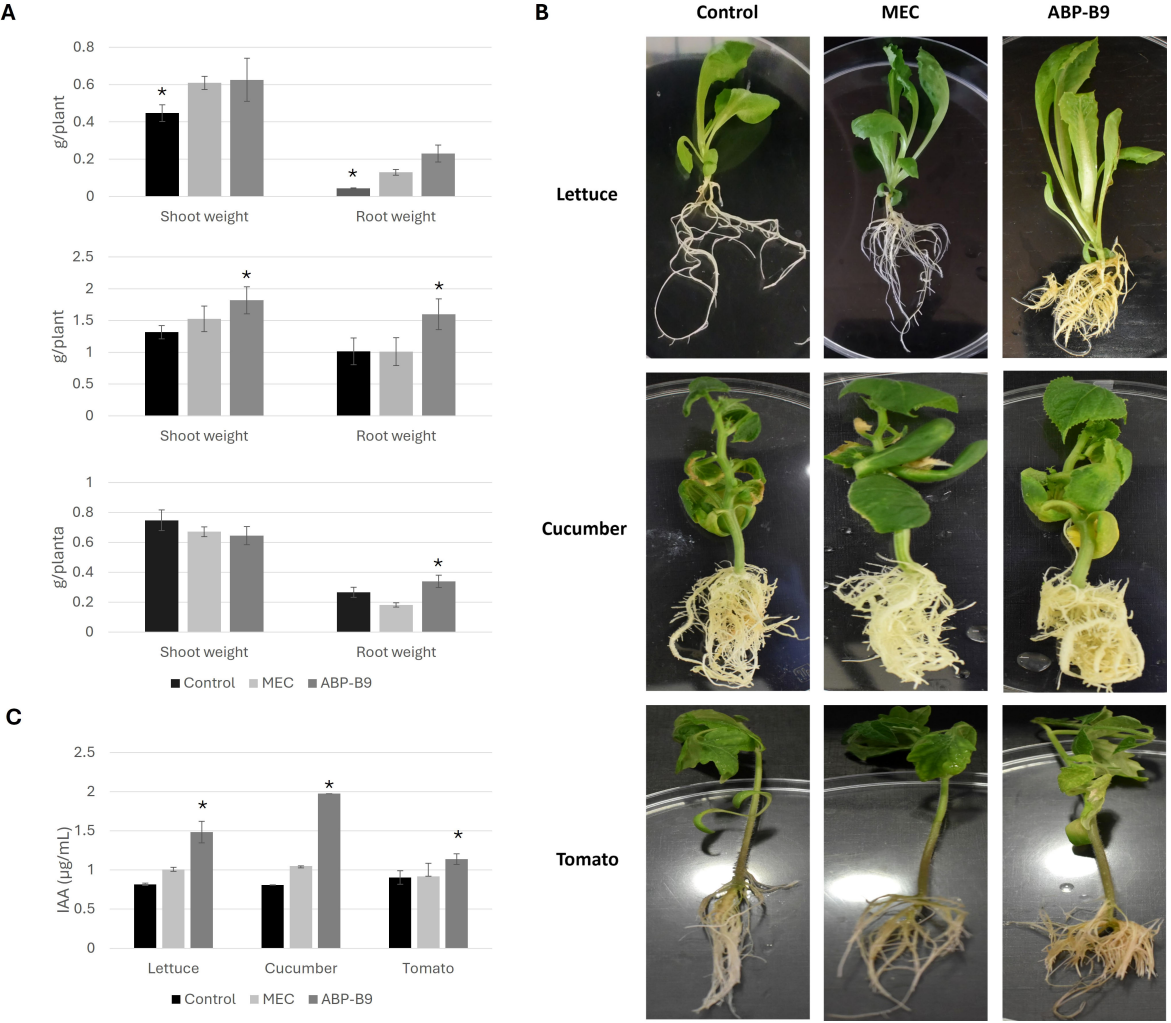
Biostimulant activity of ABP-B9 *in vitro.* **(A)** Mean shoot and root weights of 10 *in vitro*-grown lettuce, cucumber, and tomato plantlets. Asterisks indicate significant differences between treatments according to a One-Way ANOVA statistical test (p < 0.05). **(B)** Morphology of lettuce, cucumber and tomato plants grown *in vitro* showing that ABP-B9 application increased the production of secondary roots. **(C)** Amount of IAA detected in the growth medium of lettuce, cucumber, and tomato plants grown under *in vitro* conditions in the absence of ABP-B9 (control), the presence of ABP-B9-inoculated media for 48 h after which the bacteria were removed by centrifugation (MEC), and presence of ABP-B9 (ABP-B9).

**Figure 4:**
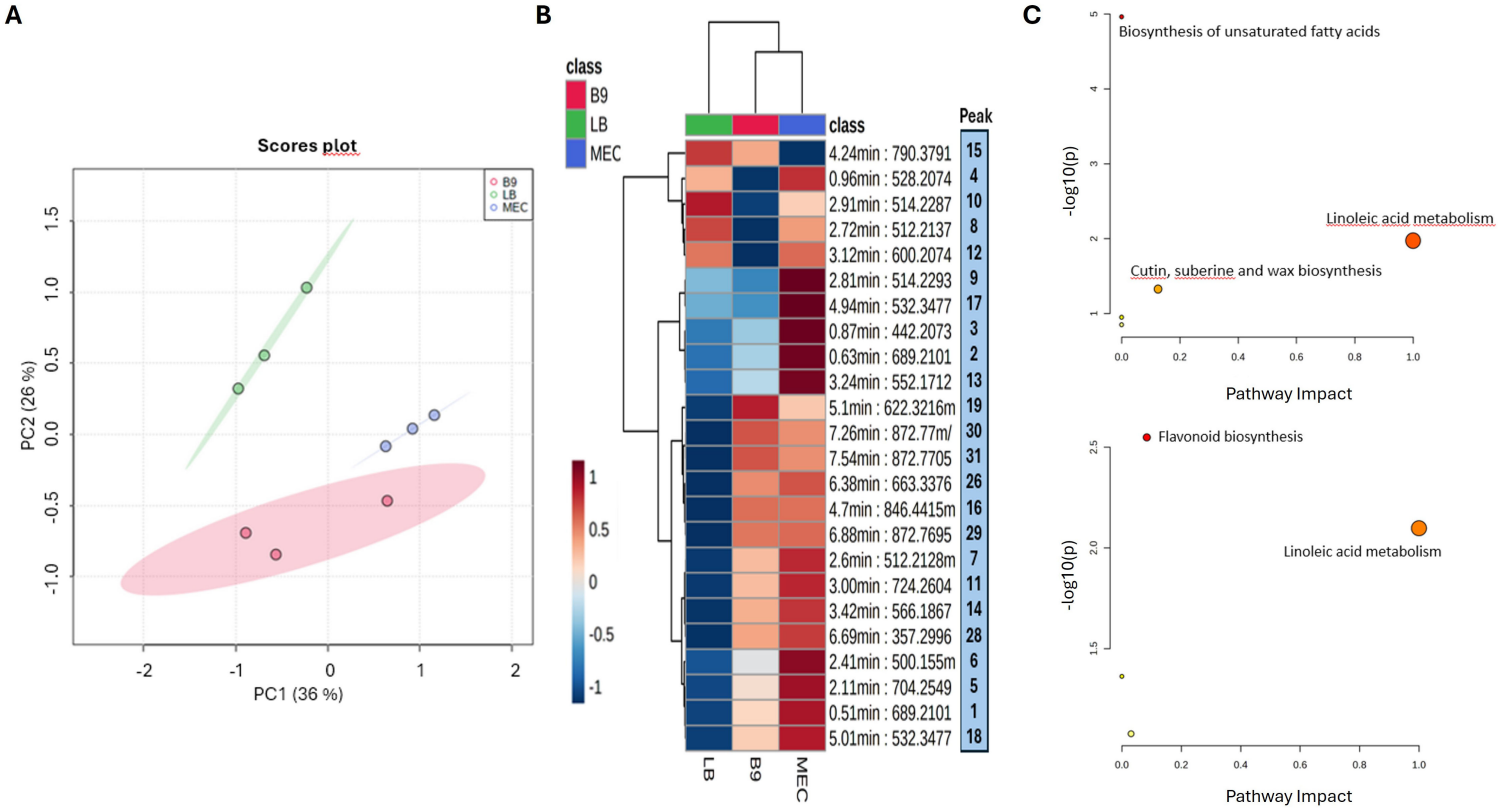
Metabolomic analysis of lettuce roots treated with ABP-B9. **(A)** PLS-DA and **(B)** hierarchical clustering heatmap of the average samples from lettuce roots. B9: roots in presence of ABP-B9; LB: roots in the absence of ABP-B9; MEC: roots in the presence of the culture broth in which ABP-B9 was grown for 48h and then removed. In **(B)**, the peak column shows the candidate compounds based on their molecular formula. **(C)** Metabolic pathways analysis. The upper image shows the most affected metabolic pathways considering all differentially expressed mass features, while the lower image shows the most affected metabolic pathways induced by the presence of B9 and MEC.

We also analyzed the changes induced in the development of lettuce, cucumber and tomato plants growing in vitro and treated with ABP-B9 (B9) or ABP-B9 media depleted of bacteria (MEC), as compared to untreated plants (Control) (**Figures 3A, B**). Plants treated with ABP-B9 exhibited a greater development of the aerial part than untreated plants, except in the case of tomato, where no significant differences were observed when compared to control plants (**Figure 3A**). This increase was also noted in the weight of the root system of the plants, which was above that of the untreated plants (**Figure 3A**). Plants treated with ABP-B9 showed decreased primary root length and a higher number of lateral roots. This effect was also observed in seedlings growing in the presence of the culture broth of isolate ABP-B9 from which the bacteria had been removed, although it was less pronounced (**Figures 3A, B**). An increase in the IAA content was observed when ABP-B9 was added to the medium in which the lettuce, tomato, and cucumber seedlings were developed, with values of 0.7, 0.2 and 1.2 µg/mL, respectively, significantly higher than control treatments (**Figure 3C**). A metabolomic analysis using three samples per treatment (LB, B9 and MEC) was carried out (**Figure 4**). A PLS-DA analysis provided two principal components explaining approximately 30% of the variation within the sample’s datasets. A clear separation between the treatments was observed, with B9 and MEC loadings grouped more closely (**Figure 4A**). According to the heatmap analysis, clearly defined patterns were observed among treatments, with the MEC-treated roots showing an up-regulation of most of the differently expressed mass features (**Figure 4B**). The list of candidate compounds (based on their molecular formula) for each detected peak was submitted to a pathway analysis, by which we observed that unsaturated fatty acids metabolism, including linolenic acid, was significantly affected in both B9 and MEC-treated roots. Other affected pathways were “Cutin, suberin and wax biosynthesis” and “Flavonoid biosynthesis” (**Figure 4C**).

### 3.3 Effect of ABP-B9 on lettuce plants’ photosynthesis performance

**Figure 5:**
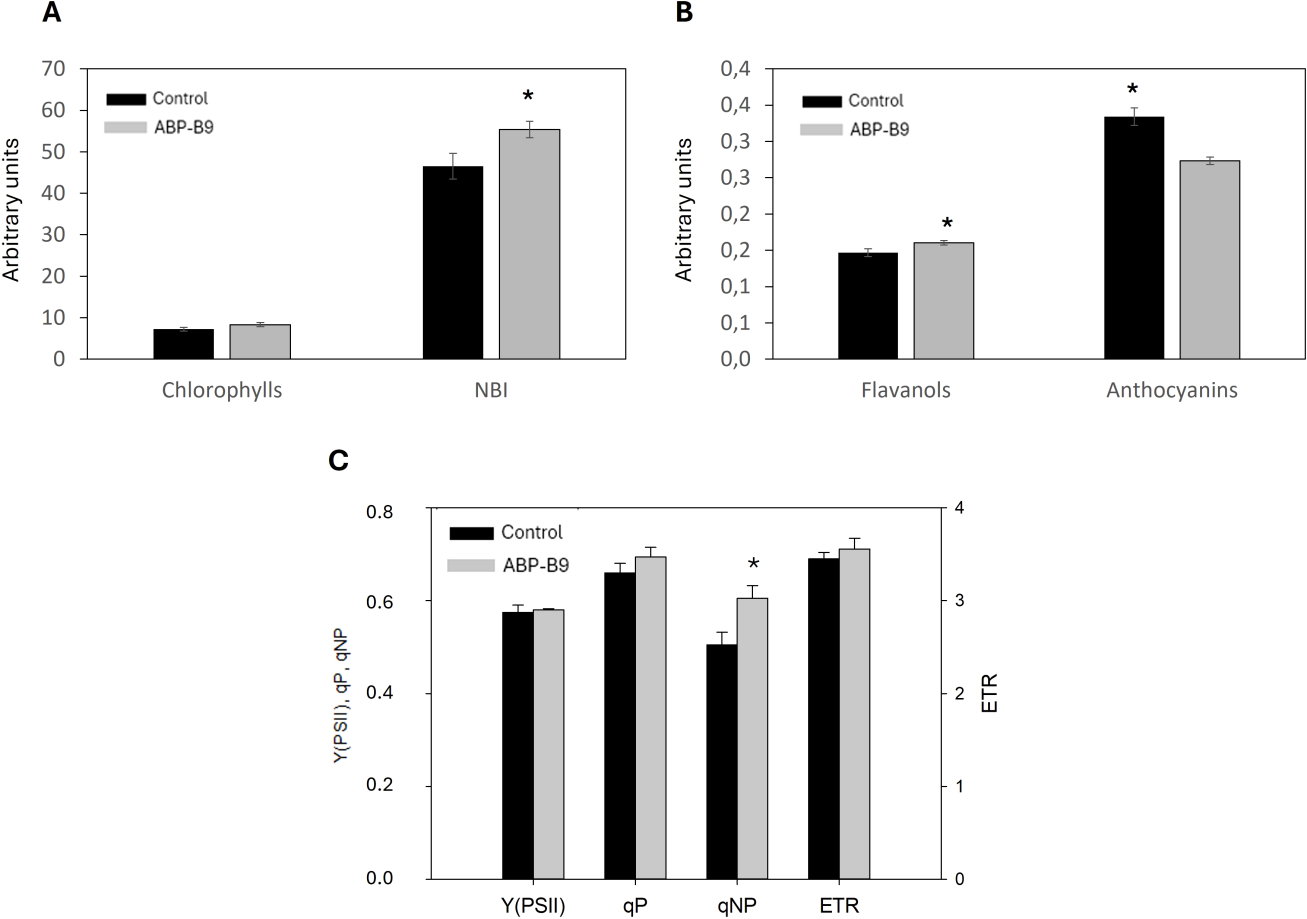
Photosynthetic performance of lettuce treated with ABP-B9. **(A)** Average values of chlorophylls and the nitrogen balance index (NBI, chlorophyll/flavonols ratio), **(B)** polyphenols (flavonoids and anthocyanins), and **(C)** fluorescence parameters, analyzed in lettuce plants of the Amenas variety grown in open field commercial cultivation, treated with ABP-B9 or untreated (control). Data are expressed as arbitrary units. Asterisks indicate significant differences between treatments according to a One-Way ANOVA statistical test (p < 0.05). The absence of asterisks indicates the lack of statistical significance.

The effect of ABP-B9 application on chlorophylls, flavonoids, and anthocyanins levels, as well as in the Nitrogen Balance Index (NBI) (**Figure 5**), was studied in an independent experiment carried out in open lettuce fields. Compared to control plants, the application of isolate ABP-B9 significantly increased flavonols levels and the NBI and decreased the levels of anthocyanins (**Figures 5A, B**), suggesting that ABP-B9 enhances the photosynthetic performance of lettuce plants, improving their nitrogen status and increasing their antioxidant capacity; this, in turn, could improve the plant’s response to stressful situations. Moreover, the analysis of fluorescence parameters performed on lettuce plants treated with ABP-B9 showed a slight increase in photochemical quenching parameters [Y(PSII) and qP] and a significant rise in the non-photochemical quenching parameter qNP (**Figure 5C**). The photochemical quenching parameters are associated to PSII efficiency, whereas qNP is related to the safe dissipation of excess energy as heat. Taken together, these data suggest a higher photosynthetic efficiency in ABP-B9-treated plants compared to untreated plants.

In summary, the enhanced photosynthetic performance, along with increased flavonoid and NBI levels in lettuce plants, suggest that the application of ABP-B9 may improve plant tolerance to environmental stress conditions.

### 3.4 Agronomic performance of crops treated with ABP-B9

The effect of ABP-B9 application on commercial crops of lettuce, celery, and spinach was evaluated in three field trials conducted in the 2021-2023 period at different locations in the Region of Murcia (**Table 2**). The results showed that the application of ABP-B9 was associated with a significant increase in the development of the lettuce treated seedlings. This effect was assessed and confirmed in lettuce five days after application. In the case of celery, although a visual improvement was observed, no quantitative evaluation was conducted. (**Table 2**; **Supplementary Figures S2**, **S3**). Analyses conducted after harvesting revealed that the application of ABP-B9 increased the development of the aerial part of the treated plants (**Table 2**). In lettuce, a significant increase in head weight and size was observed (**Table 2**). In celery, plants of a greater length were obtained, while the application of ABP-B9 to spinach plants resulted in a significant increase in plant weight and length (**Table 2**). An analysis of the root systems revealed that in all cases, the application of ABP-B9 led to a significant increase in root dry weight (**Table 2**). Consistently, an evaluation of the presence of ABP-B9 in the root system of treated plants at the end of the crop cycle revealed that it remained alive throughout the crop cycle with 10^3^ - 10^5^ CFU/plant (**Table 2**). These data are consistent with studies conducted under in vitro conditions, where the application of ABP-B9 increased the production of secondary roots (see above). Consequently, the application of ABP-B9 resulted in an increase in crop yields, with the yield increasing by 22% (**Table 2**).

**Table 2:** Field assays description and summary of agronomic results obtained from each crop evaluated after the application of ABP-B9.

Field assays description						
Crop	Lettuce		Celery		Spinach	
Variety	Amenas		PH535		El Giga	
Number of plants per treatment	150		100		2100	
Type of crop	Open field		Open field		Open field	
Location	Garrobillo-Murcia		Águilas-Murcia		Fuente Álamo - Cartagena	
Transplant date	24/11/2023		14/10/2021		30/12/2021	
End of cultivation	13/02/2024		3/02/2022		22/02/2022	
Modality	Conventional		Conventional		Conventional	
No. of applications	1		1		1	
Application time	In seedbed 5 days before transplantation		In seedbed 5 days before transplantation		20 days after sowing in the field	
Application dose	1.7 mL/seedling (≥1 x 10^7^ UFC/mL)		1.7 mL/seedling (≥1 x 10^7^ UFC/mL)		1.7 mL/seedling (≥1 x 10^7^ UFC/mL)	

Agronomic results						
Agronomic traits	Lettuce		Celery		Spinach	
	Control	ABP-B9	Control	ABP-B9	Control	ABP-B9
Seedling weight (g)	0.84 ± 0.02 b*	1.2 ± 0.02 a				
Seedling shoot length (cm)	5.2 ± 0.2 b	6.15 ± 0.1 a				
Plant weight (g)	605.3 ± 13.2 b	663.1 ± 12.6 a	1336.9 ± 25.5	1380.6 ± 30.8	5.4 ± 0.2 b	6.0 ± 0.2 a
Bud weight (g)	332.9 ± 8,7 b	392.7 ± 8.8 a				
Radial diameter (cm)	12.6 ± 0,1 b	13.2 ± 0.1 a				
Pole diameter (cm)	11.6 ± 0.1 b	12.2 ± 0.1 a				
Length (cm)			93.2 ± 0.5 b	99.1 ± 0.6 a	11.1 ± 0.1 b	11.9 ± 0.1 a
Root dry weight (g)	2.7 ± 0.1 b	3,4 ± 0.1 a	4.6 ± 0.2 b	5.7 ± 0.2 a	24.9 ± 0.8 b	28.1 ± 0.9 a
Yield (Kg/ha)	23287.5 b	28453.2 a	126215.9	132234. 6	36064.7	38526.03
Colony-forming units of ABP-B9 (UFC/g root)	2.7 x10^3^ ± 6.9 x10^2^ b	6.3 x10^3^ ± 1.6x10^3^ a	1.4x10^4^ ± 8.9x10^2^	1.1 x10^4^ ± 6.7 x10^3^	8.4 x10^3^ ± 1.0 x10^3^b	1.7 x10^5^ ± 4.2 x10^4^ a

*Different letters within the same row, for each crop, denote significant statistical differences according to the One-Way ANOVA or t-test (p<0.05). The absence of letters indicates no significant differences.

Furthermore, it was observed that the application of ABP-B9 to lettuce crops reduced weight loss by 16% (P=0.017; P>0.05) and decreased head rot during cold storage, as only mild symptoms of rot were observed in some of the 10 analyzed lettuce heads (**Supplementary Figure S4**). These findings suggest that ABP-B9 can increase the shelf life of harvested lettuce.

### 3.5 ABP-B9 genome sequencing and taxonomic placement

**Figure 6:**
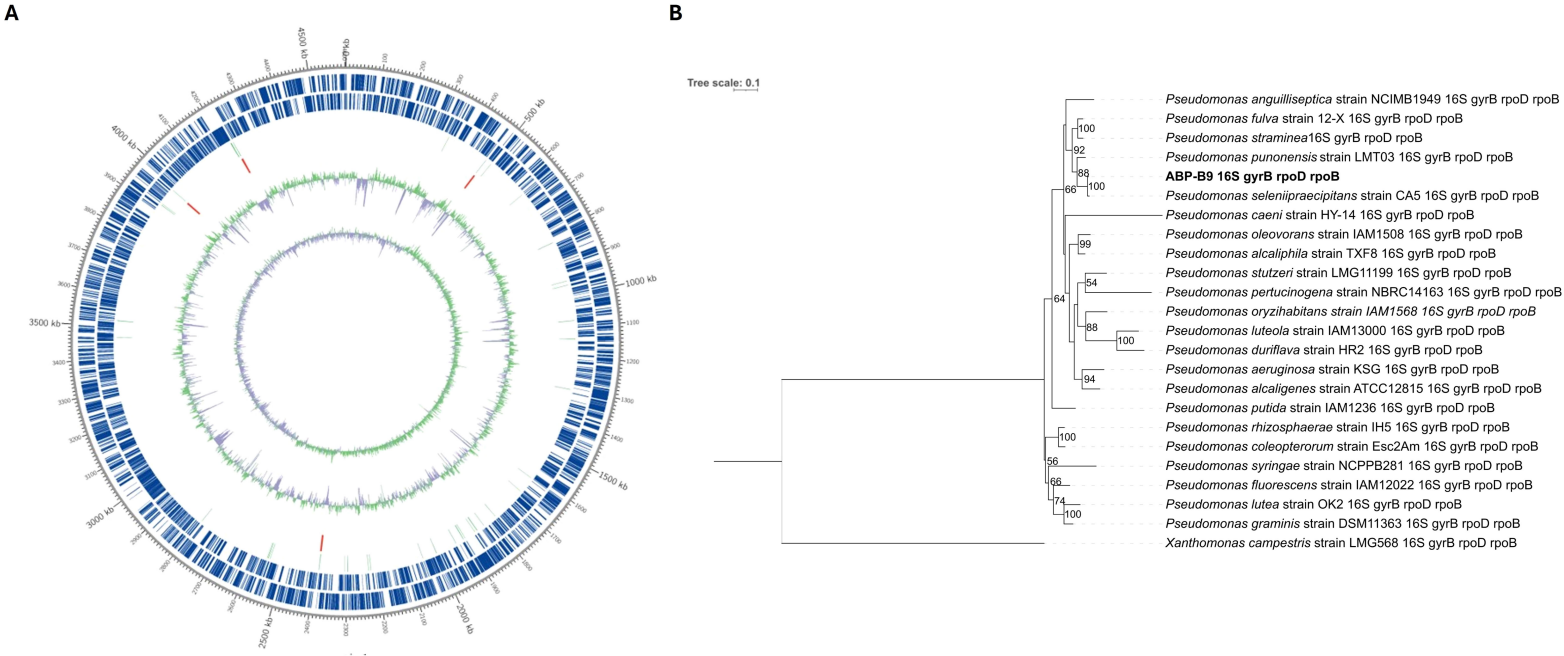
Genomic and phylogenetic analyses of ABP-B9. **(A)** A circular map representing the ABP-B9 genome. Marked features are shown from outside to the center. The forward CDS represents the region of forward coding sequences (CDS), with non-CDS regions indicated as blank. The reverse CDS indicates the region of reverse coding sequences (CDS), also marked as blank where not applicable. The tRNA region is highlighted in light green, while the rRNA region is marked in red. The GC content is represented with regions above the average GC percentage shown in an outer light green peak, and those below average indicated in an inner lavender peak; the peak height reflects the difference from the average GC percentage. The GC skew is calculated using the formula (G-C)/(G+C), where a positive value indicates G dominance and a negative value indicates C dominance. **(B)** Phylogenetic analysis of ABP-B9 and different *Pseudomonas* species. The evolutionary history was inferred by using the Maximum Likelihood method based on the General Time Reversible model with Gamma-distributed rate variation across sites and a proportion of Invariant sites (GTR+G+I). The analysis involved 24 nucleotide sequences of concatenated partial sequences of the housekeeping genes 16S rRNA, *gyrB, rpoD*, and *rpoB*. All positions containing gaps and missing data were eliminated. There was a total of 2,776 positions in the final alignment. The tree with the highest log likelihood is shown. The tree is drawn to scale, with branch lengths measured in the number of substitutions per site. The percentage of trees (bootstrap) in which the associated taxa clustered together is shown next to the branches. Only bootstrap values higher than 50% were displayed. *Xanthomonas campestris* strain LMG568 was used as an outgroup.

Whole genome sequencing using the PacBio platform produced a total of 11,484,944 reads with an average length of 4,602,102 bp and a genome coverage depth of about 161. The complete genome sequence was 4,602,210 bp with a GC content of 61.46%. The genome contains 4,325 predicted genes, 4,247 coding sequences (CDSs), 66 tRNAs, and 12 rRNAs. The ABP-B9 genome is circular (**Figure 6A**). The number of genes associated with general Clusters of Orthologous Groups (COG) functional categories is shown in **Table 3**. Biological roles were assigned to 3,137 (74.13%) genes of the predicted coding sequences based on similarity searches. The remaining coding sequences (1,095) were classified as proteins with an unknown function (**Table 3**).

**Table 3:** Number of ABP-B9 genes associated with general EggNOG functional categories.

EggNOG Code^*^	Description	Count	Ratio (%)
J	Translation, ribosomal structure and biogenesis	171	4.0406
A	RNA processing and modification	1	0.0236
K	Transcription	251	5.9310
L	Replication, recombination and repair	150	3.5444
B	Chromatin structure and dynamics	2	0.0473
D	Cell cycle control, cell division, chromosome partitioning	35	0.8270
Y	Nuclear structure	0	0.0000
V	Defense mechanisms	38	0.8979
T	Signal transduction mechanisms	244	5.7656
M	Cell wall/membrane/envelope biogenesis	219	5.1749
N	Cell motility	97	2.2921
Z	Cytoskeleton	0	0.0000
W	Extracellular structures	0	0.0000
U	Intracellular trafficking, secretion, and vesicular transport	80	1.8904
O	Posttranslational modification, protein turnover, chaperones	140	3.3081
C	Energy production and conversion	231	5.4584
G	Carbohydrate transport and metabolism	182	4.3006
E	Amino acid transport and metabolism	346	8.1758
F	Nucleotide transport and metabolism	66	1.5595
H	Coenzyme transport and metabolism	120	2.8355
I	Lipid transport and metabolism	120	2.8355
P	Inorganic ion transport and metabolism	274	6.4745
Q	Secondary metabolites biosynthesis, transport and catabolism	63	1.4887
R	General function prediction only	307	7.2543
S	Function unknown	1095	25.8743
Total	-	4232	100

*Coding and functional annotation provided by the Evolutionary genealogy of genes: Non-supervised Orthologous Groups (EggNOG) database.

An analysis of housekeeping gene sequences (*16S rRNA, gyrB, rpoD, and rpoB*), both individually and concatenated, showed a percentage identity greater than 98% with strains of *P. seleniipraecipitans* (**Supplementary Table S3**). The identity percentages exceeded the discrimination thresholds established by Mulet et al. (2010), allowing for the classification of ABP-B9 as a strain of *P. seleniipraecipitans*. Genome-based methods for species delimitation were also employed to infer the taxonomic affiliation of ABP-B9. The TETRA, ANIm, ANIb and dDDH indices were calculated for the three genomes showing the highest scores on the housekeeping gene analysis above. When ABP-B9 was compared with *P. seleniipraecipitans* LMG 25475[T], the values obtained exceeded the established species thresholds, which varies from 70% to 94-96%, depending on the parameter used. When ABP-B9 was compared with *P. fulva* 12-X and *P. punonensis* CECT 8089[T], these values were below thresholds (**Table 4**).

**Table 4:** Genome-based indices for species delimitation when comparing ABP-B9 with the highest similarity strains of *P. seleniipraecipitans*, *P. fulva* and *P. punonensis*.

*Pseudomonas* strain	ANIb^a^	ANIm^a^	TETRA^a^			dDDH^b^				G+C difference	AAI^c^	Ortho ANIb^d^
	(>95% same strain)	(>95% same strain)	(>99 same species)	Formula 1		Formula 2		Formula 3			(≥ 95-96%)	(≥ 95-96%)
	%	%	z-score	DDH	Prob. DDH >= 70%	DDH	Prob. DDH >= 70%	DDH	Prob. DDH >= 70%	%		
*P. fulva* 12-X	83.61	86.53	0.97361	54.20	22.61	27.50	0.03	45.90	0.91	2.05	0.93	84.08
*P. seleniipraecipitans* LMG 25475 [T]	97.92	98.27	0.99976	91.20	97.69	84.00	93.18	92.60	99.69	0.05	0.99	98.24
*P. punonensis* CECT 8089 [T]	83.60	86.54	0.99255	58.00	34.83	27.50	0.03	48.50	1.86	0.36	0.92	

a (http://jspecies.ribohost.com/jspeciesws/).

b (http://ggdc.dsmz.de; Meier-Kolthoff et al., 2013).

c http://ekhidna2.biocenter.helsinki.fi/AAI/.

d https://www.ezbiocloud.net/tools/ani.

A phylogenetic analysis was performed from the alignments of concatenated sequences of the *16S rRNA*, *gyr*B, *rpo*D, and *rpo*B genes from different *Pseudomonas* species, showing a consistent phylogenetic assignment of ABP-B9 to a known species, group, or subgroup (**Figure 6B**). ABP-B9 was placed in the same phylogenetic branch as other *P. seleniipraecipitans* isolates, with a bootstrap value of 100%. Phylogeny and genome comparison analyses suggested that ABP-B9 is included in the *P. fluorescens* lineage, belonging to the phylogenetic group of *P. straminea* (Peix et al., 2018) (**Figure 6**; **Table 4**).

### 3.6 Genome mining

We then analyzed the ABP-B9 genome to identify genes or gene clusters that may be responsible for producing novel or bioactive compounds, such as antibiotics, enzymes, or secondary metabolites. Using the ICEfinder tool, we found one integrative and conjugative element (ICE) of 20 kb from position 24,390 to 44,235 in the bacterial genome. It had a GC content of 62.98% (-1.48% with respect to the genome average; **Supplementary Figure S5**). This ICE encodes 22 proteins comprising a likely functional Type IV secretion system (T4SS) apparatus with a TraJ protein and plasmid transfer ATPase TraJ, both involved in bacterial conjugation, specifically in the transfer of plasmids from one bacterium to another. The genome analysis of ABP-B9 using the SecReT6 server (https://bioinfo-mml.sjtu.edu.cn/SecReT6/t6ss_prediction.php) confirmed the absence of T6SS in the genome. This result is consistent with the findings from the RAST analysis (see below), which also detected the presence of a TSS4, comprising 17 subsystems. Using the ICEfinder tool, no integrated plasmids were detected, and no Origin of Transfer sequences (oriT) was found (**Supplementary Figure S5**).

One intact 55.8 kb-prophage of the *Pseudomonas* Dobby and phiCTX family was identified between positions 2,744,746 and 2,800,628 using the PHASTER software. The prophage has a GC content of 60.32% (**Supplementary Figure S6**). Importantly, the ctx gene encoding a eukaryotic cell pore-forming toxin, originally described for the phiCTX phage of *P. aeruginosa* hosts (Hayashi et al., 1990), is not present in the prophage region of ABP-B9 (**Supplementary Figure S6**). A second but incomplete prophage region is present between positions 2,619,235 and 26,360,140 (16.9 kb; **Supplementary Figure S6**), and it most likely represents a phage remnant. Clustered Regularly Interspaced Short Palindromic Repeats (CRISPR) elements were not found. ABP-B9 does not seem to produce mycotoxins, according to the results obtained from the analysis with the ToxFinder 1.0 tool.

**Figure 7:**
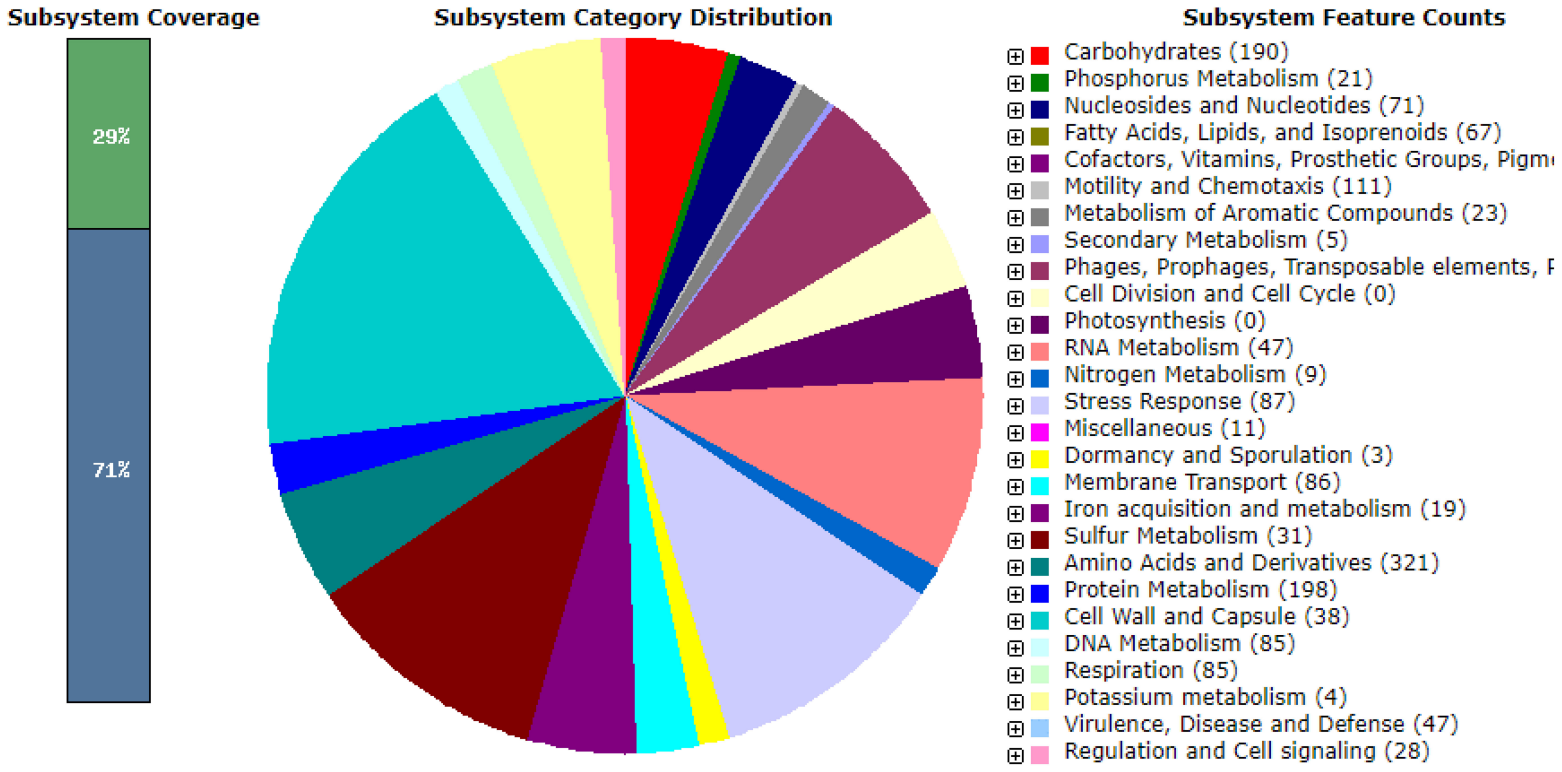
Subsystem categories detected in the ABP-B9 genome using the RAST annotation server (https://rast.nmpdr.org/).

A RAST analysis showed that 29% of the ABP-B9 genes are associated with known subsystems, while 71% remain unclassified in the subsystem coverage (**Figure 7**). The category with the greatest number of predicted genes was amino acid metabolism and derivatives, with 321 genes, followed by protein metabolism and carbohydrates with 198 and 190 genes, respectively (**Figure 7**). Additionally, the analysis featured 66 enzymes involved in metabolic pathways related to biosynthesis of plant hormones, 48 involved in the biosynthesis of phenylpropanoids, 10 involved in the fatty acid biosynthesis, and 10 involved in terpenoid biosynthesis (**Supplementary Table S4**). We detected the presence of genes involved in promoting plant growth, including genes involved in the synthesis of trehalose, IAA, urease, nitric oxide, ammonium assimilation, and phosphate solubilization (**Supplementary Table S4**). Furthermore, the 1-aminocyclopropane-1-carboxylate (ACC) deaminase enzyme was also identified (**Supplementary Table S4**). Moreover, ABP-B9 shows a potential for producing volatile metabolites such as acetoin and 2,3-butanediol (**Supplementary Table S4**).

We used two tools, RAST and AntiSMASH, to identify genes responsible for the bio-stimulatory capacity, given the demonstrated effects produced by the bacterium. ABP-B9 lacks genes related to the synthesis of specific polysaccharides such as *pel* and *psl*, and only the presence of the genes *algB, algC*, and *algE* was detected. The RAST analysis showed that the ABP-B9 genome includes genes involved in the production of capsules and extracellular polysaccharides (22 genes), of which 11 are related to rhamnose-containing glycans, seven to dTDP-rhamnose synthesis, and four to exopolysaccharide biosynthesis. Glycans may play a role in surface adherence, biofilm formation, and interaction with plants (**Supplementary Table S5**), which is consistent with some of our experimental observations (**Figure 3**). ABP-B9 does not seem to have the genes required to produce pyoverdine, pyochelin, achromobactin, or bacillibactin, but it does have genes involved in the synthesis, transport and export of enterobactin, and receptors for pyoverdine, pyochelin, anguibactin, and pseudobactin (**Supplementary Table S5**). The ABP-B9 genome does not encode alkaline proteases or chitinases.

An AntiSMASH analysis was performed to detect biosynthetic gene clusters (BGCs) potentially responsible for producing biostimulant agents. ABP-B9 contains five distinct BGCs in its genome, primarily associated with ribosomally synthesized and post-translationally modified peptides (RiPPs), N-acetylglutaminylglutamine amide dipeptide (NAGGN), as well as other chemical compounds such as betalactone, terpene, arylpolyene, and resorcinol (**Supplementary Table S6**; **Supplementary Figure S8**). Little to no similarity was observed between the identified molecules and previously reported clusters (ranging from 21% to 40% similarity). The terpene cluster showed the highest similarity, with a 100% similarity to a known carotenoid; the arylpolyene and resorcinol cluster showed a 40% similarity to APE Vf (aryl polyene cluster), while NAGGN showed a 21% similarity to O-antigen (**Supplementary Table S6**). Two of these clusters did not match any known clusters closely, corresponding to the RiPP-like (region 1) and betalactone (region 2) types.

According to the PathogenFinder tool of the Center for Genomic Epidemiology (Cosentino et al., 2013), the isolate ABP-B9 genome corresponds to a microorganism that is non-pathogenic to humans (**Supplementary Figure S7**). Accordingly, an analysis with VFDB of the presence of virulence factors showed the absence of hits in the ABP-B9 genome. To experimentally validate this observation, an acute oral toxicity study according to guide OECD 423:2001 showed that ABP-B9 was non-pathogenic in a murine model following oral inoculation of 1.95 mL/Kg (1 × 10^8^ UFC/mL).

Additional genome analyses showed that ABP-B9 could exhibit heavy metal resistance, as several heavy metal resistance genes were identified in its genome (**Supplementary Table S7**). ABP-B9 could potentially survive in environments with high concentrations of nickel, copper, cadmium, zinc, molybdate, cobalt, arsenate and chromate. Specifically, four genes potentially involved in nickel resistance, 15 genes in copper resistance, four in cadmium resistance, 16 in zinc resistance, four in molybdate resistance, 10 genes in cobalt resistance, six in arsenate resistance, and one in chromate resistance, were identified in the ABP-B9 genome (**Supplementary Table S7**). Consistently, our RAST analysis showed ABP-B9 subsystems related to tolerance/resistance to these heavy metals.

## 4 Discussion

The use of biofertilizers and biostimulants based on microorganisms contributes towards more sustainable agricultural practices and can also increase the resilience of agricultural systems. Despite the significant advantages that *Pseudomonas*-based biostimulants could offer to agriculture (Schwanemann et al., 2020; Pieterse et al., 2021), their effective implementation faces several challenges that must be addressed through scientific research and technological development. The identification and characterization of new isolates of the genus *Pseudomonas* with biostimulant activities may help exploit the full potential of this genus for developing more efficient microorganism-based products.

Our study presents and describes ABP-B9, a representative of a new strain of *Pseudomonas seleniipraecipitans*, isolated from the rhizosphere of lettuce plants. ABP-B9 showed several interesting features that can be of agricultural interest, particularly its biostimulant ability. The study of the ABP-B9 isolate revealed significant insights into its taxonomic classification, genomic characteristics, and potential agricultural benefits. ABP-B9 has typical phenotypic traits of the *Pseudomonas* genus (**Table 1**; **Supplementary Figure S1**). Phylogenetic and genomic analyses positioned isolate ABP-B9 within the *P. fluorescens* lineage, closely related to other sequenced isolates of *P. seleniipraecipitans* (98% identity). Whole-genome comparisons further confirmed the correct species assignment thus we concluded that isolate ABP-B9 represents a novel strain of *Pseudomonas seleniipraecipitans*, which we named *P. seleniipraecipitans* strain ABP-B9.

The isolate most closely related to ABP-B9 described in the literature is *P. seleniipraecipitans* strain CA5, which showed 99% identity in the analysis of housekeeping gene sequences (**Supplementary Table S2**). CA5 is resistant to high concentrations of both selenate and selenite, and could be useful as an inoculum for bioreactors used to harvest selenium from selenite-containing groundwater due to its ability to reduce selenite to elemental red selenium (Hunter and Manter, 2009; Hunter, 2014). There is limited research on *P. seleniipraecipitans* as a PGPB, and research on other species of the genus has been mostly focused on *P. fluorescens* isolates, which have been proven to play an important role in antifungal activity (Ganeshan and Manoj Kumar, 2005; Petatán-Sagahón et al., 2011). Rocha et al. (2016) detected plant growth-promoting traits and enzymatic activities in two isolates of *P. seleniipraecipitans*, PA100 and PA35. Both isolates showed a 99% identity with isolate ABP-B9 (**Supplementary Table S2**). These bacteria could produce IAA, siderophores, and HCN, and solubilize phosphate (**Table 1**; **Supplementary Table S5**). While these isolates did not exhibit antimicrobial activity against the pathogens tested *in vitro*, they were able to produce lytic enzymes (Rocha et al., 2016). Isolate PA100 produced protease, lipase, cellulase, pectinase, amylase, and xylanase, whereas isolate PA35 produced cellulase, pectinase, amylase, and xylanase. The analysis of the ABP-B9 genome suggested that it only produces lipase and amylase (**Supplementary Table S5**). Ajijah et al. (2023) showed the distribution of genes potentially involved in plant growth promotion in the genome of *P. seleniipraecipitans* D1-6. This isolate exhibited traits related to siderophores, IAA and gamma-aminobutyric acid (GABA) synthesis (Ajijah et al., 2023).

Under field conditions, the treatment of lettuce, celery, and spinach plants with liquid media containing ABP-B9 increased yields by 22%, 5%, and 7%, respectively (**Table 2**). There are at least two effects of ABP-B9 that can contribute to explain yield improvements shared by the three crop species; these are the increase in the development of the root systems, and the enhancement of the photosynthetic performance of the plants. The application of ABP-B9 led to an increase in the emergence of secondary roots, an effect that we also observed in *in vitro* tests (**Figure 3B**). Secondary root development can facilitate nutrient uptake by the plant and its establishment in the field after transplantation (Ortíz-Castro et al., 2009; Pérez-Rodriguez et al., 2020). The increase in root system development could be attributed to the IAA produced by ABP-B9 (**Figure 3C**), which plays crucial roles in cell division, differentiation, germination, control of vegetative growth, and the synthesis of pigments and secondary metabolites (Nathan et al., 2017). Indeed, our genome analysis revealed the presence of the genes *trpA, trpB, trpC, trpD, trpE, trpF, trpG, trpL* and *trpS*, involved in the production of IAA (**Supplementary Table S5**), indicating that the biosynthesis of IAA is tryptophan-dependent, where tryptophan is used as a precursor (Tang et al., 2023). It has been reported that *Pseudomonas* strains produce higher levels of IAA than other beneficial bacteria (Xie et al., 1996). Additionally, it has been observed that redox-active compounds such as thioredoxins and glutaredoxins are important in the control of root development (Alloing et al., 2018; Cantabella et al., 2020). In the genome of ABP-B9, we observed the presence of genes involved in the synthesis of three types of both glutaredoxins and thioredoxins (**Supplementary Table S5**). On the other hand, our metabolomic study of the lettuce seedlings’ root systems showed that both the presence of ABP-B9 and MEC (culture broth without ABP-B9) significantly affected the metabolism of unsaturated fatty acids and linoleic acid, which are important components of membranes (**Figure 4C**). This may suggest that ABP-B9 may modulate the physicochemical properties of root cell membranes. Also, the “Flavonoid biosynthesis” pathway was induced in lettuce roots by both the presence of ABP-B9 and MEC (**Figure 4C**); phenylpropanoid-related compounds are key players in plant growth and development, with enhanced phenylpropanoid metabolism leading to increased development (Barba Espin et al., 2024).

ABP-B9 enhanced the photosynthetic performance of plants, improving their nitrogen balance status and increasing their antioxidant capacity (**Figure 5**), which in turn can improve plant responses to stressful situations (Jurado-Mañogil et al., 2023). This improvement in the photosynthetic state of the plants is likely due to enhanced nutrient uptake and increased iron availability, an essential micronutrient in various processes such as photosynthesis and chlorophyll synthesis. In this sense, ABP-B9 produces siderophores that chelate iron and make it available to the plant, and it can also facilitate nitrogen availability by converting atmospheric nitrogen into ammonium or nitrates (**Table 1**; **Supplementary Table S5**). Other studies have described increased chlorophyll content and photoprotective compounds in plants treated with *Pseudomonas* spp (Pérez-Rodriguez et al., 2020; Ghadamgahi et al., 2022).

The biostimulant effect of the isolate was observed five days after its application at the seedling stage, resulting in a significant increase in the development of treated seedlings, both in the aerial part and the root system (**Table 2**; **Supplementary Figures S2**, **S3**). The fact that the biostimulant effect is observed in such a short time may be associated with the efficient colonization of the root system by ABP-B9. Efficient plant growth stimulation requires effective root colonization, which often relies on bacterial cell surface structures such as pili. Type IV pili are complex protein systems that allow bacteria to produce pili, with their function controlled by numerous genes. A total of 17 genes involved in type IV pili biosynthesis were identified in the genome of ABP-B9 (**Supplementary Table S5**).

Another remarkable result is the increased shelf life observed in harvested lettuce treated with ABP-B9 (**Supplementary Figure S4**). ABP-B9’s capacity to produce substances with antioxidant activity, such as carotenoids and arylpolyenes (Schöner et al., 2016), or those related to protection against oxidative stress such as ACC and trehalose, which diminish plant ethylene levels (Stout and Nüsslein, 2010), could have a significant impact on the shelf life of harvested lettuce. Antioxidants are crucial for neutralizing free radicals and reducing cell oxidative stress, which is particularly important during post-harvesting.

Finally, genes that contribute to the environmental adaptation of ABP-B9 were also identified in its genome, including those responsible for heavy metal resistance such as nickel, copper, cadmium, zinc, molybdate, cobalt, arsenate, sulphur, and chromate (**Supplementary Table S7**). ABP-B9 is also capable of using a wide variety of compounds as carbon sources, including glycerol, acetate, cyanophycin, cellobiose, acetoin, and GABA. This potential resistance and the ability of the isolate to utilize a wide range of carbon sources demonstrate its capacity to thrive in adverse and contaminated environments, by employing specific mechanisms to counteract the toxic effects of these elements.

## 5 Conclusion

ABP-B9, a new rhizospheric isolate of lettuce belonging to the species *Pseudomonas seleniipraecipitans*, exhibits significant potential as a biostimulant. Given the genetic evaluation and tests on murines, there is no evidence that it is pathogenic for humans, thus, its use in agriculture appears to be safe. ABP-B9’s abilities to enhance crop growth and improve yield, along with its safety profile, makes it a valuable addition to the sustainable agricultural practices toolbox.

*In vitro* and genomic traits have provided valuable information that will allow performing a functional characterization of the mechanisms of action of ABP-B9 in the field and developing a formulation that maintains the characteristics of the isolate and its viability during storage. Additionally, the genetic analysis revealed the presence of genes indicating potential bacterial properties that have yet to be characterized.

## Data Availability

The original contributions presented in the study are publicly available. This data can be found here: Genbank, CP194332.

## Supplementary material

Supplementary Figure 1 Results of the bioMérieux API 20 NE Gallery System for ABP-B9 and identification based on APIWEB™.

Supplementary Figure 2 Images of lettuce crop development under commercial production conditions. **(A)**, **(B)**, and **(C)** show the development of lettuce seedlings five days after inoculation with ABP-B9 at the time of field transplantation. ABP-B9 (treated with ABP-B9) and control (untreated). **(D)**, **(E)**, **(F)**, and **(G)** depict the crop’s evolution.

Supplementary Figure 3
**(A)** Images of celery crop development under commercial production conditions. Upper images show the development of seedlings inoculated with the ABP-B9 isolate (ABP-B9) or untreated (control) five days before field transplantation, and lower images depict the crop’s evolution. **(B)** Images of spinach crop development under commercial production conditions.

Supplementary Figure 4
**(A)** Visual scale used to assess the severity of lettuce rot during cold storage for up to 15 days. The scale ranges from mild (1), moderate (2), severe (3) and advanced rot (4). Images of storage damage in lettuce treated with ABP-B9 (ABP-B9) and untreated (control) lettuce after 16 days at 4°C are also shown (bottom pictures on the right). **(B)** Mean weight loss of 10 head lettuces after sixteen days of storage at 4°C. Asterisks indicate significant differences between treatments according to a One-Way ANOVA statistical test (p < 0.05).

Supplementary Figure 5 Identification of an integrative and conjugative element (ICE) in the genome of ABP-B9 with the ICEfinder tool (https://bioinfo-mml.sjtu.edu.cn/ICEfinder/index.php).

Supplementary Figure 6 Identification of prophage regions in the genome of ABP-B9. Prophago loci were identified with the Phaster tool (https://phaster.ca/).

Supplementary Figure 7 Output of PathogenFinder tool from the Center for Genomic Epidemiology (https://cge.food.dtu.dk/services/PathogenFinder/) for the analysis of the ABP-B9 genome.

Supplementary Figure 8 Representation of each secondary metabolite biosynthetic gene cluster in the genome of ABP-B9, predicted by antiSMASH. RiPP-like, Ribosomally synthesized and post-translationally modified Peptide; Betalactone; Terpene- carotenoid; NAGGN, N-acetylglutaminylglutamine amide dipeptide; Arylpolyene-APE Vf; resorcinol.

## References

[B1] Ajijah N. Fiodor A. Dziurzynski M. Stasiuk R. Pawlowska J. Dziewit L.. (2023). Biocontrol potential of *Pseudomonas protegens* ML15 against Botrytis cinerea causing gray mold on postharvest tomato (Solanum lycopersicum var. cerasiforme). Front. Plant Sci. 14. doi: 10.3389/fpls.2023.1288408 PMC10748600 38143572

[B2] Alloing G. Mandon K. Boncompagni E. Montrichard F. Frendo P. (2018). Involvement of glutaredoxin and thioredoxin systems in the nitrogen-fixing symbiosis between legumes and rhizobia. Antioxidants 7 (12), 182. doi: 10.3390/antiox7120182 30563061 PMC6315971

[B3] Altschul S. F. Madden T. L. Schäffer A. A. Zhang J. Zhang Z. Miller W.. (1997). Gapped BLAST and PSI-BLAST: a new generation of protein database search programs. Nucleic Acids Res. 25, 3389–3402. doi: 10.1093/nar/25.17.3389 9254694 PMC146917

[B4] Arndt D. Grant J. R. Marcu A. Sajed T. Pon A. Liang Y.. (2016). PHASTER: a better, faster version of the PHAST phage search tool. Nucleic Acids Res. 44, W16–W21. doi: 10.1093/nar/gkw387 27141966 PMC4987931

[B5] Bakker A. W. Schippers B. O. B. (1987). Microbial cyanide production in the rhizosphere in relation to potato yield reduction and *Pseudomonas* spp-mediated plant growth-stimulation. Soil Biol. Biochem. 19, 451–457. doi: 10.1016/0038-0717(87)90037-X

[B6] Barba Espin G. Díaz-Vivancos P. Pérez-Caselles C. Faize L. Hernandez J. Pedreño M.. (2024). Tomato plants expressing a stilbene synthase gene display genotype-depending alterations in metabolome profile and antioxidant system. Physiol. Plant 176 (1), e14147. doi: 10.1111/ppl.14147

[B7] Blin K. Shaw S. Kloosterman A. M. Charlop-Powers Z. van Wezel G. P. Medema M. H.. (2021). antiSMASH 6.0: improving cluster detection and comparison capabilities. Nucleic Acids Res. 49, W29–W35. doi: 10.1093/nar/gkab335 33978755 PMC8262755

[B8] Boddey R. M. Dobereiner J. (1995). Nitrogen fixation associated with grasses and cereals: Recent progress and perspectives for the future. Fertil. Res. 42, 241–250. doi: 10.1007/BF00750518

[B9] Bortolaia V. Kaas R. S. Ruppe E. Roberts M. C. Schwarz S. Cattoir V.. (2020). ResFinder 4.0 for predictions of phenotypes from genotypes. J. Antimicrob. Chemother. 75, 3491–3500. doi: 10.1093/jac/dkaa345 32780112 PMC7662176

[B10] Brock A. K. Berger B. Mewis I. Ruppel S. (2013). Impact of the PGPB Enterobacter radicincitans DSM 16656 on growth, glucosinolate profile, and immune responses of Arabidopsis thaliana. Microb. Ecol. 65, 661–670. doi: 10.1007/s00248-012-0146-3 23242136

[B11] Cantabella D. Karpinska B. Teixido N. Dolcet-Sanjuan R. Foyer C. (2020). Regulation of root architecture by *Pseudomonas* oryzihabitans is mediated by strigolactones and redox processes. Authorea. doi: 10.22541/au.159819295.56193824

[B12] Chin C.-S. Alexander D. H. Marks P. Klammer A. A. Drake J. Heiner C.. (2013). Nonhybrid, finished microbial genome assemblies from long-read SMRT sequencing data. Nat. Methods 10, 563–569. doi: 10.1038/nmeth.2474 23644548

[B13] Cosentino S. Voldby Larsen M. Møller Aarestrup F. Lund O. (2013). PathogenFinder–distinguishing friend from foe using bacterial whole genome sequence data. PloS One 8, e7730. doi: 10.1371/journal.pone.0077302 PMC3810466 24204795

[B14] Coutinho T. Franco G. Lobo F. (2015). Homology-independent metrics for comparative genomics. Comput. Struct. Biotechnol. J. 6, (352-7). doi: 10.1016/j.csbj.2015.04.005 PMC4446528 26029354

[B15] Doyle J. (1991). “DNA Protocols for Plants BT”, in. Hewitt G. M. Johnston A. W. B. Young J. P. W. (eds.) Molecular Techniques in Taxonomy (Springer Berlin Heidelberg, Berlin, Heidelberg), 283–293. doi: 10.1007/978-3-642-83962-7_18

[B16] Drogue B. Doré H. Borland S. Wisniewski-Dyé F. Prigent-Combaret C. (2012). Which specificity in cooperation between phytostimulating rhizobacteria and plants? Res. Microbiol. 163, 500–510. doi: 10.1016/j.resmic.2012.08.006 22989671

[B17] FAOSTAT (2021). FAOSTAT statistical database. Food and Agriculture Organization of the United Nations. Available online at: https://www.fao.org/faostat/es/#data/TP

[B18] Felsenstein J. (1981). Evolutionary trees from DNA sequences: A maximum likelihood approach. J. Mol. Evol. 17, 368–376 doi: 10.1007/BF01734359 7288891

[B19] Gamborg O. L. Miller R. A. Ojima K. (1968). Nutrient requirements of suspension cultures of soybean root cells. Exp. Cell Res. 50, 151–158. doi: 10.1016/0014-4827(68)90403-5 5650857

[B20] Ganeshan G. Manoj Kumar A. (2005). *Pseudomonas fluorescens*, a potential bacterial antagonist to control plant diseases. J. Plant Interact. 1, 123–134. doi: 10.1080/17429140600907043

[B21] Ghadamgahi F. Tarighi S. Taheri P. Saripella G. V. Anzalone A. Kalyandurg P. B.. (2022). Plant Growth-Promoting Activity of *Pseudomonas aeruginosa* FG106 and Its Ability to Act as a Biocontrol Agent against Potato, Tomato and Taro Pathogens. Biol. (Basel). 11 (1), 140. doi: 10.3390/biology11010140 PMC8773043 35053136

[B22] Gordon S. A. Weber R. P. (1951). COLORIMETRIC ESTIMATION OF INDOLEACETIC ACID. Plant Physiol. 26, 192–195. doi: 10.1104/pp.26.1.192 16654351 PMC437633

[B23] Granja G. Fernanda M. L. (2010).Aislamiento e identificación de microorganismos solubilizadores de potasio a partir de muestras de suelo y raíces de cultivos de alcachofa de la localidad de la remonta, cantón Cayambe. Available online at: https://api.semanticscholar.org/CorpusID:84072755 (Accessed September 1, 2023).

[B24] Grant J. R. Enns E. Marinier E. Mandal A. Herman E. K. Chen C.-Y.. (2023). Proksee: in-depth characterization and visualization of bacterial genomes. Nucleic Acids Res. 51, W484–W492. doi: 10.1093/nar/gkad326 37140037 PMC10320063

[B25] Hayashi T. Baba T. Matsumoto H. Terawaki Y. (1990). Phage-conversion of cytotoxin production in *Pseudomonas aeruginosa* . Mol. Microbiol. 4, 1703–1709. doi: 10.1111/j.1365-2958.1990.tb00547.x 2127632

[B26] Higuera S. Pavlov M. Sousa L. Vásquez-Ponce F. Parás-Silva J. Martínez J.. (2023). *Pseudomonas aquigelida sp.* nov., an Antarctic bacterium isolated from seawater of Fildes Bay, King George Island. Preprint, Research Square. doi: 10.21203/rs.3.rs-3342923/v1

[B27] Huerta-Cepas J. Szklarczyk D. Heller D. Hernández-Plaza A. Forslund S. K. Cook H.. (2019). eggNOG 5.0: a hierarchical, functionally and phylogenetically annotated orthology resource based on 5090 organisms and 2502 viruses. Nucleic Acids Res. 47, D309–D314. doi: 10.1093/nar/gky1085 30418610 PMC6324079

[B28] Hunter W. J. (2014). *Pseudomonas seleniipraecipitans* proteins potentially involved in selenite reduction. Curr. Microbiol. 69, 69–74. doi: 10.1007/s00284-014-0555-2 24604150

[B29] Hunter W. J. Manter D. K. (2009). Reduction of selenite to elemental red selenium by *Pseudomonas* sp. Strain CA5. Curr. Microbiol. 58, 493–498. doi: 10.1007/s00284-009-9358-2 19189180

[B30] Jurado C. Díaz-Vivancos P. Gregorio B.-E. Acosta-Motos J. R. Hernández J. A. (2024). Effect of halophyte-based management in physiological and biochemical responses of tomato plants under moderately saline greenhouse conditions. Plant Physiol. Biochem. 206, 108228. doi: 10.1016/j.plaphy.2023.108228 38043255

[B31] Jurado-Mañogil C. Barba-Espín G. Hernández J. A. Diaz-Vivancos P. (2023). Comparative metabolomic analysis between tomato and halophyte plants under intercropping conditions. Physiol. Plant 175, e13971. doi: 10.1111/ppl.13971 37616015

[B32] Karnwal A. (2021). Screening and identification of abiotic stress-responsive efficient antifungal *Pseudomonas* spp. From rice rhizospheric soil. BioTechnologia 102, 5–19. doi: 10.5114/bta.2021.103758 36605708 PMC9642915

[B33] Konstantinidis K. T. Tiedje J. M. (2005). Genomic insights that advance the species definition for prokaryotes. Proc. Natl. Acad. Sci. U. S. A. 102, 2567–2572. doi: 10.1073/pnas.0409727102 15701695 PMC549018

[B34] Kumar S. Stecher G. Tamura K. (2016). MEGA7: molecular evolutionary genetics analysis version 7.0 for bigger datasets. Mol. Biol. Evol. 33 (7), 1870–1874. doi: 10.1093/molbev/msw054 PMC8210823 27004904

[B35] Lalucat J. A. Bosch R. García-Valdés E. Palleroni. N. J. (2006). Biology of *Pseudomonas stutzeri* . Microbiol. Mol. Biol. Rev. 70 (2), 510–547. doi: 10.1128/mmbr.00047-05 PMC1489536 16760312

[B36] Leis E. Erickson S. Waller D. Richard J. Goldberg T. (2019). A comparison of bacteria cultured from unionid mussel hemolymph between stable populations in the upper mississippi river basin and populations affected by a mortality event in the clinch river. Freshw. Mollusk Biol. Conserv. 22, 70–80. doi: 10.31931/fmbc.v22i2.2019.70-80

[B37] Li S. Park Y. Duraisingham S. Strobel F. H. Khan N. Soltow Q. A.. (2013). Predicting network activity from high throughput metabolomics. PloS Comput. Biol. 9, e1003123. doi: 10.1371/journal.pcbi.1003123 23861661 PMC3701697

[B38] Li J. Yao Y. Xu H. H. Hao L. Deng Z. Rajakumar K.. (2015). SecReT6: a web-based resource for type VI secretion systems found in bacteria. Environ. Microbiol. 17, 2196–2202. doi: 10.1111/1462-2920.12794 25640659

[B39] Liu M. Li X. Xie Y. Bi D. Sun J. Li J.. (2019). ICEberg 2.0: an updated database of bacterial integrative and conjugative elements. Nucleic Acids Res. 47, D660–D665. doi: 10.1093/nar/gky1123 30407568 PMC6323972

[B40] Martínez-Sánchez M. d. l. Á. Martínez-Hernández G. B. López-Gómez A. (2024). Extending more than one week the shelf life of fresh-cut lettuce using vinegar enriched in bioactive compounds encapsulated in α-cyclodextrins. Foods 13 (19), 3142. doi: 10.3390/foods13193142 39410177 PMC11475928

[B41] Medlar A. J. Törönen P. Holm L. (2018). AAI-profiler: fast proteome-wide exploratory analysis reveals taxonomic identity, misclassification and contamination. Nucleic Acids Res. 46, W479–W485. doi: 10.1093/nar/gky359 29762724 PMC6030964

[B42] Meier-Kolthoff J. P. Auch A. F. Klenk H.-P. Göker M. (2013). Genome sequence-based species delimitation with confidence intervals and improved distance functions. BMC Bioinf. 14, 60. doi: 10.1186/1471-2105-14-60 PMC3665452 23432962

[B43] Mulet M. Lalucat J. García-Valdés E. (2010). DNA sequence-based analysis of the *Pseudomonas* species. Environ. Microbiol. 12, 1513–1530. doi: 10.1111/j.1462-2920.2010.02181.x 20192968

[B44] Murashige T. Skoog F. (2006). A revised medium for rapid growth and bio assays with tobacco tissue cultures. Physiol. Plant 15, 473–497. doi: 10.1111/j.1399-3054.1962.tb08052.x

[B45] Nathan V. Rajam K. Rani M. (2017). Plant growth promotion efficacy of indole acetic acid (IAA) produced by a mangrove associated fungi-trichoderma viride VKF3. Int. J. Curr. Microbiol. App. Sci. 6 (11), 2692–2701. doi: 10.20546/ijcmas.2017.611.xxx

[B46] Navarro J. A. Botella F. Maruhenda A. Sastre P. Sánchez-Pina M. A. Pallas V. (2004). Comparative infection progress analysis of lettuce big-vein virus and mirafiori lettuce virus in lettuce crops by developed molecular diagnosis techniques. Phytopathology 94, 470–477. doi: 10.1094/PHYTO.2004.94.5.470 18943765

[B47] Ortíz-Castro R. Contreras-Cornejo H. A. Macías-Rodríguez L. López-Bucio J. (2009). The role of microbial signals in plant growth and development. Plant Signal. Behav. 4, 701–712. doi: 10.4161/psb.4.8.9047 19820333 PMC2801380

[B48] Parte A. C. Sardà Carbasse J. Meier-Kolthoff J. P. Reimer L. C. Göker M. (2020). List of Prokaryotic names with Standing in Nomenclature (LPSN) moves to the DSMZ. Int. J. Syst. Evol. Microbiol. 70, 5607–5612. doi: 10.1099/ijsem.0.004332 32701423 PMC7723251

[B49] Peix A. Ramírez-Bahena M. H. Velázquez E. (2018). The current status on the taxonomy of *Pseudomonas* revisited: An update. Infect. Genet. Evol. 57, 106–116. doi: 10.1016/j.meegid.2017.10.026 29104095

[B50] Pérez-Rodriguez M. Pontin M. Lipinski V. Bottini R. Piccoli P. Cohen A. (2020). *Pseudomonas fluorescens* and *Azospirillum brasilense* Increase Yield and Fruit Quality of Tomato Under Field Conditions. J. Soil Sci. Plant Nutr. 20, 1–11. doi: 10.1007/s42729-020-00233-x

[B51] Petatán-Sagahón I. Anducho-Reyes M. Silva-Rojas H. Arana A. Tellez A. Cárdenas-Álvarez I.. (2011). Isolation of Bacteria with Antifungal Activity against the Phytopathogenic Fungi Stenocarpella maydis and Stenocarpella macrospora. Int. J. Mol. Sci. 12, 5522–5537. doi: 10.3390/ijms12095522 22016606 PMC3189730

[B52] Pieterse C. Berendsen R. de Jonge R. Stringlis I. Dijken A. van Pelt J. A.. (2021). *Pseudomonas simiae* WCS417: star track of a model beneficial rhizobacterium. Plant Soil 461, 245–263. doi: 10.1007/s11104-020-04786-9

[B53] Richter M. Rosselló-Móra R. Oliver Glöckner F. Peplies J. (2016). JSpeciesWS: a web server for prokaryotic species circumscription based on pairwise genome comparison. Bioinformatics 32, 929–931. doi: 10.1093/bioinformatics/btv681 26576653 PMC5939971

[B54] Rocha J. Tacão M. Fidalgo C. Alves A. Henriques I. (2016). Diversity of endophytic *Pseudomonas* in Halimione portulacoides from metal(loid)-polluted salt marshes. Environ. Sci. Pollut. Res. Int. 23, 13255–13267. doi: 10.1007/s11356-016-6483-x 27023813

[B55] Rodríguez-Blanco A. Sicardi M. Frioni L. (2015). Plant genotype and nitrogen fertilization effects on abundance and diversity of diazotrophic bacteria associated with maize (Zea mays L.). Biol. Fertil. Soils 51, 391–402. doi: 10.1007/s00374-014-0986-8

[B56] Schöner T. A. Gassel S. Osawa A. Tobias N. J. Okuno Y. Sakakibara Y.. (2016). Aryl polyenes, a highly abundant class of bacterial natural products, are functionally related to antioxidative carotenoids. Chembiochem 17, 247–253. doi: 10.1002/cbic.201500474 26629877

[B57] Schreiter S. Ding G. Grosch R. Kropf S. Antweiler K. Smalla K. (2014). Soil type dependent effects of a potential biocontrol inoculant on indigenous bacterial communities in the rhizosphere of field-grown lettuce. FEMS Microbiol. Ecol. 90 (3), 718–30. doi: 10.1111/1574-6941.12430 25244497

[B58] Schwanemann T. Otto M. Wierckx N. Wynands B. (2020). *Pseudomonas* as versatile aromatics cell factory. Biotechnol. J. 15, e1900569. doi: 10.1002/biot.201900569 32978889

[B59] Seemann T. (2014). Prokka: rapid prokaryotic genome annotation. Bioinformatics 30, 2068–2069. doi: 10.1093/bioinformatics/btu153 24642063

[B60] Stout L. Nüsslein K. (2010). Biotechnological potential of aquatic plant-microbe interactions. Curr. Opin. Biotechnol. 21, 339–345. doi: 10.1016/j.copbio.2010.04.004 20494570

[B61] Tang J. Li Y. Zhang L. Mu J. Jiang Y. Fu H.. (2023). Biosynthetic pathways and functions of indole-3-acetic acid in microorganisms. Microorganisms 11 (8), 2077. doi: 10.3390/microorganisms11082077 37630637 PMC10459833

[B62] Tatusova T. DiCuccio M. Badretdin A. Chetvernin V. Nawrocki E. P. Zaslavsky L.. (2016). NCBI prokaryotic genome annotation pipeline. Nucleic Acids Res. 44, 6614–6624. doi: 10.1093/nar/gkw569 27342282 PMC5001611

[B63] Thompson J. D. Gibson T. J. Plewniak F. Jeanmougin J. Higgins D. G. (1997). The ClustalX windows interface: flexible strategies for multiple sequence alignment aided by quality analysis tools. Nucleis Acids Res. 25 (24), 4876–4882. doi: 10.1093/nar/25.24.4876 PMC147148 9396791

[B64] Turner S. Pryer K. M. Miao V. P. W. Palmer J. D. (1999). Investigating deep phylogenetic relationships among cyanobacteria and plastids by small subunit rRNA sequence analysis. J. Eukaryot. Microbiol. 46, 327–338. doi: 10.1111/j.1550-7408.1999.tb04612.x 10461381

[B65] Wang M. Goh Y.-X. Tai C. Wang H. Deng Z. Ou H.-Y. (2022). VRprofile2: detection of antibiotic resistance-associated mobilome in bacterial pathogens. Nucleic Acids Res. 50. doi: 10.1093/nar/gkac321 PMC9252795 35524563

[B66] Widawati S. Suliasih S. (2019). Role of indigenous nitrogen-fixing bacteria in promoting plant growth on post tin mining soil. Makara J. Sci. 23, 28–38. doi: 10.7454/mss.v23i1.10801

[B67] Xie H. Pasternak J. J. Glick B. (1996). Isolation and characterization of mutants of the plant growth-promoting rhizobacterium *Pseudomonas putida* GR12-2 that overproduce indoleacetic acid. Curr. Microbiol. 32, 67–71. doi: 10.1007/s002849900012

[B68] Yoon S.-H. Ha S.-M. Lim J. Kwon S. Chun J. (2017). A large-scale evaluation of algorithms to calculate average nucleotide identity. Antonie Van Leeuwenhoek 110, 1281–1286. doi: 10.1007/s10482-017-0844-4 28204908

[B69] Zhou Y. Liang Y. Lynch K. H. Dennis J. J. Wishart D. S. (2011). PHAST: a fast phage search tool. Nucleic Acids Res. 39, W347–W352. doi: 10.1093/nar/gkr485 21672955 PMC3125810

