# Supplementary figures and images for: ABP-B9, a new strain of Pseudomonas seleniipraecipitans with biostimulant activity
Source: Front Plant Sci. 2025 Jun 25;16:1561298. doi: 10.3389/fpls.2025.1561298 (PMC12239753; doi:10.3389/fpls.2025.1561298)

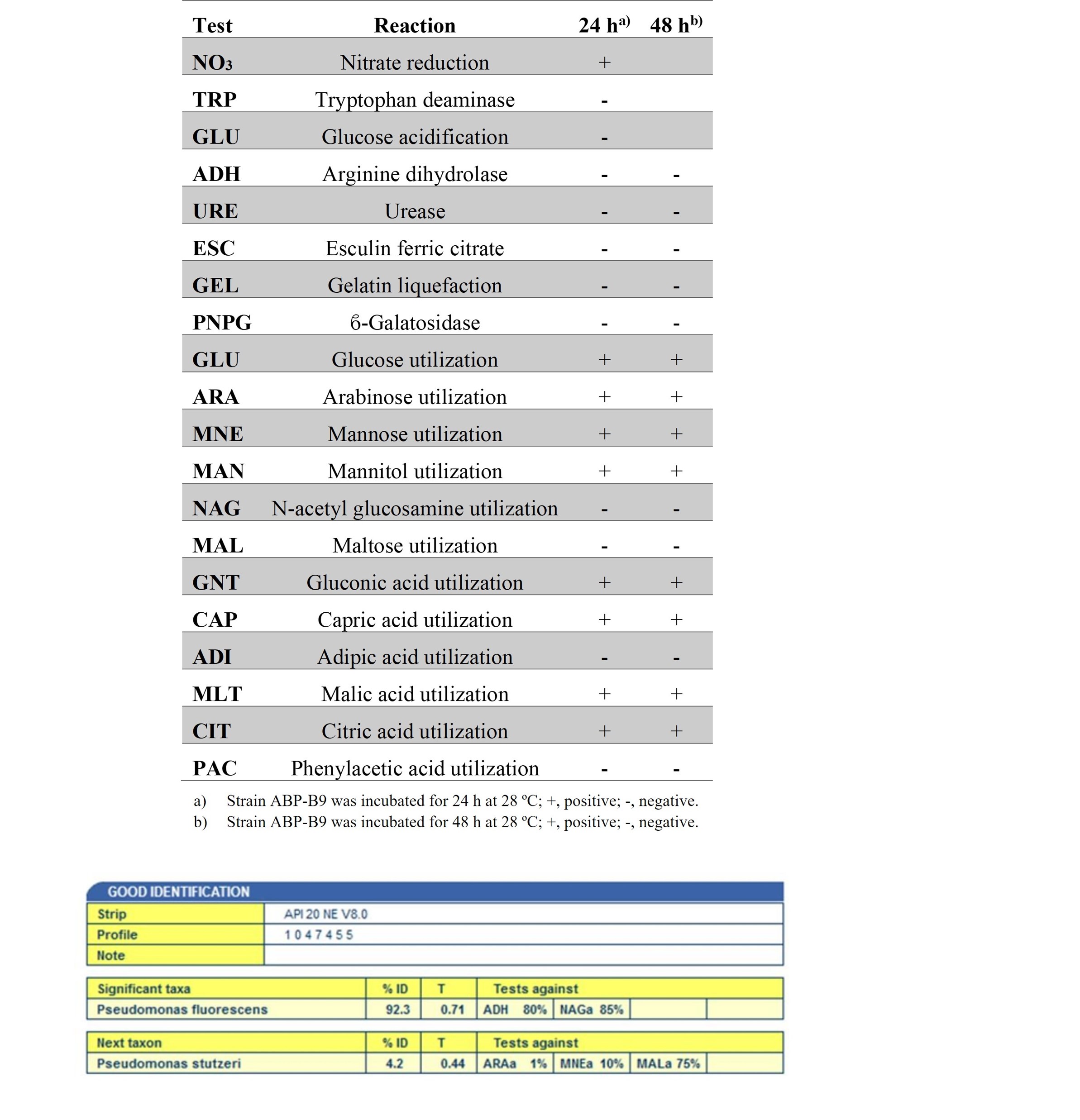

Supplement: Supplementary Figure 1 — Results of the bioMérieux API 20 NE Gallery System for ABP-B9 and identification based on APIWEB™. [file Image1.jpeg]

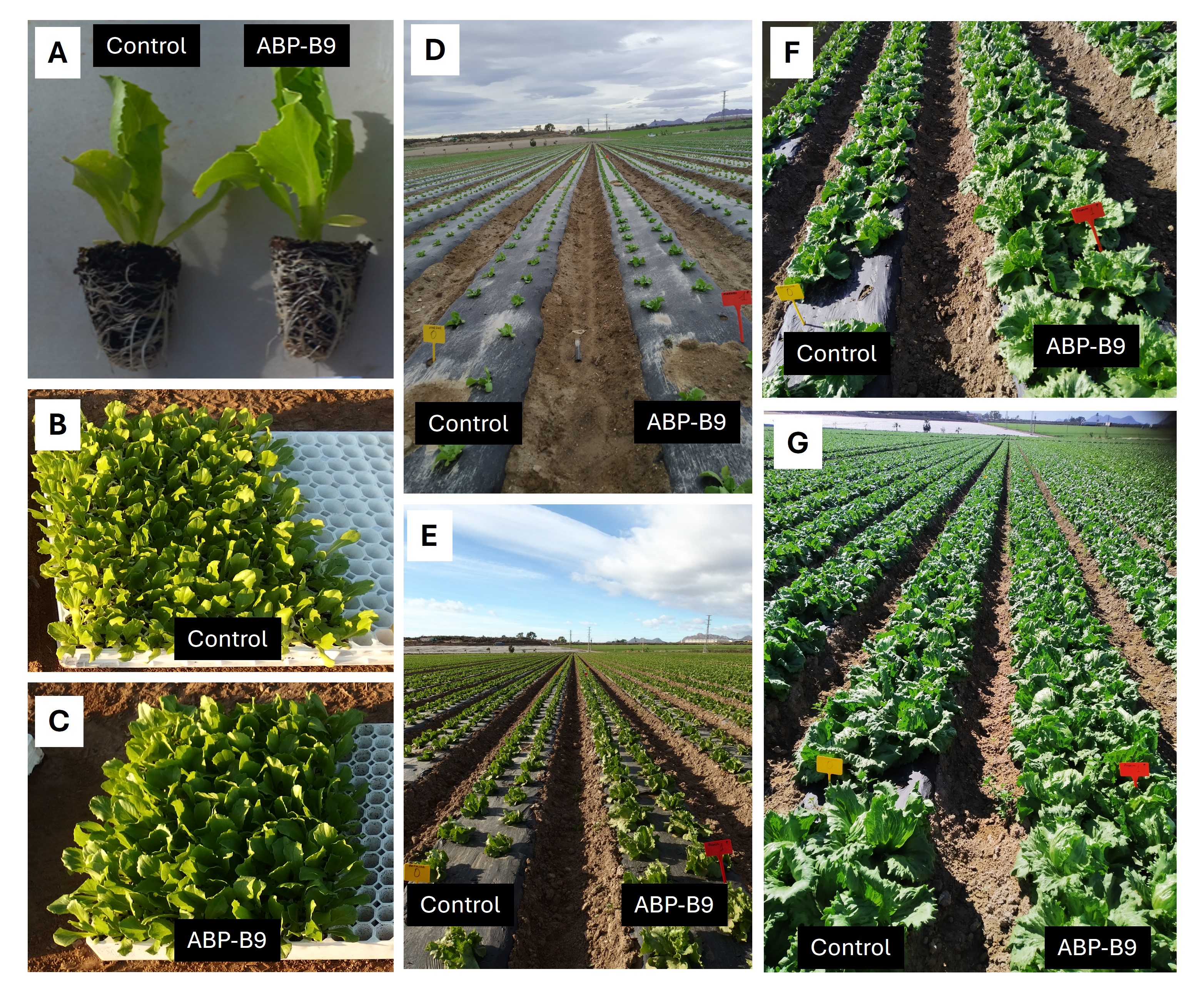

Supplement: Supplementary Figure 2 — Images of lettuce crop development under commercial production conditions. (A), (B), and (C) show the development of lettuce seedlings five days after inoculation with ABP-B9 at the time of field transplantation. ABP-B9 (treated with ABP-B9) and control (untreated). (D), (E), (F), and (G) depict the crop’s evolution. [file Image2.jpeg]

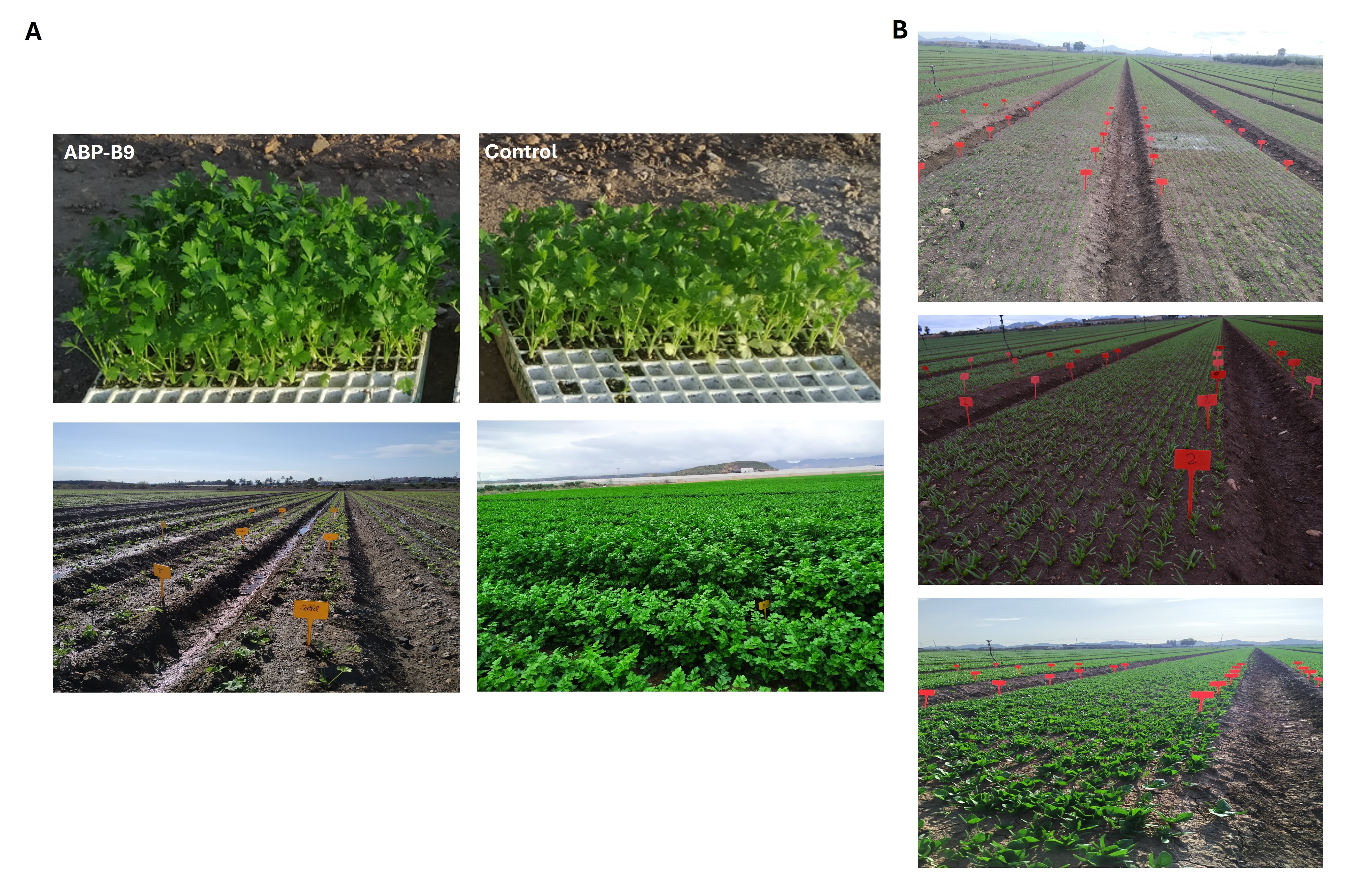

Supplement: Supplementary Figure 3 — (A) Images of celery crop development under commercial production conditions. Upper images show the development of seedlings inoculated with the ABP-B9 isolate (ABP-B9) or untreated (control) five days before field transplantation, and lower images depict the crop’s evolution. (B) Images of spinach crop development under commercial production conditions. [file Image3.jpeg]

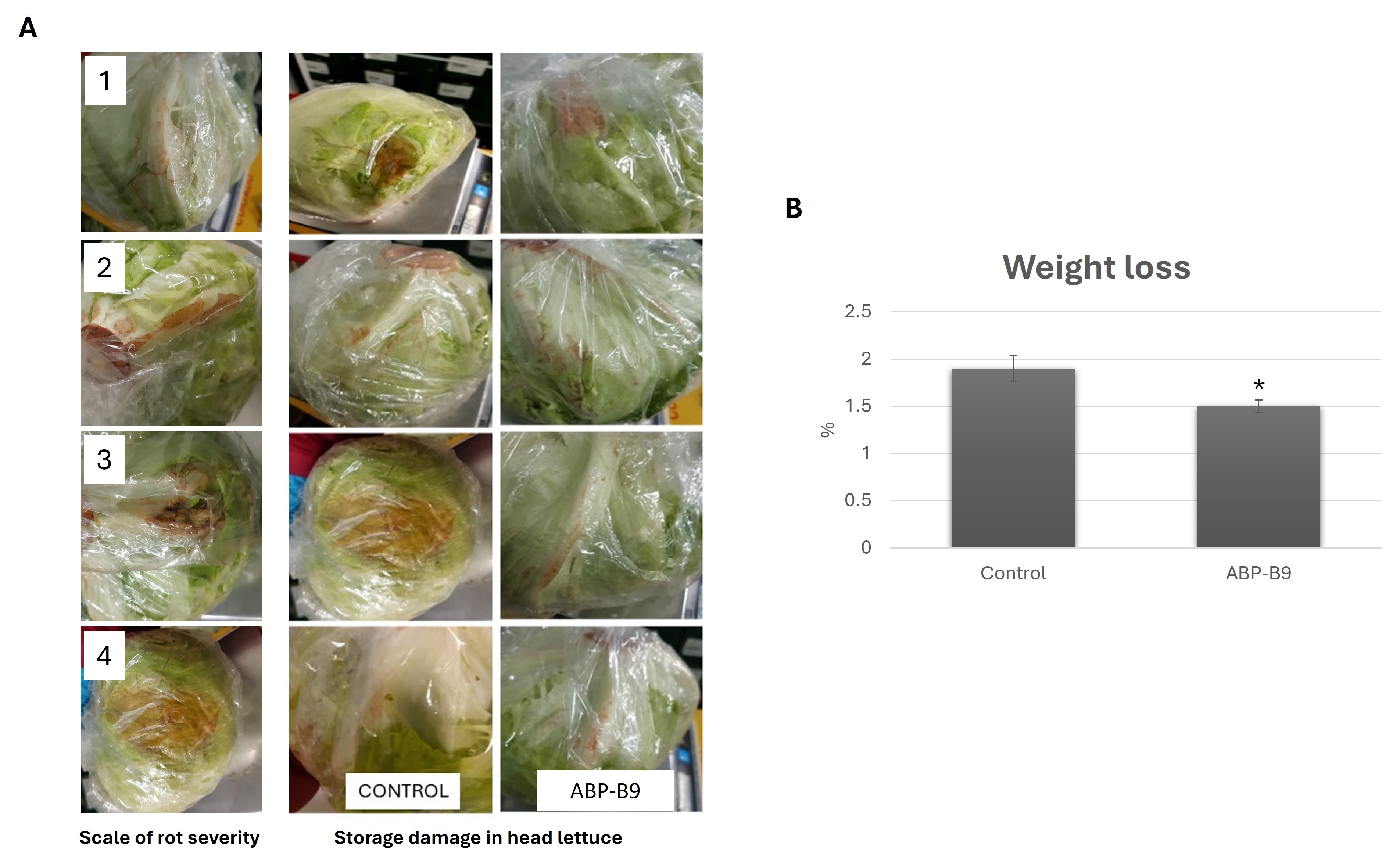

Supplement: Supplementary Figure 4 — (A) Visual scale used to assess the severity of lettuce rot during cold storage for up to 15 days. The scale ranges from mild (1), moderate (2), severe (3) and advanced rot (4). Images of storage damage in lettuce treated with ABP-B9 (ABP-B9) and untreated (control) lettuce after 16 days at 4°C are also shown (bottom pictures on the right). (B) Mean weight loss of 10 head lettuces after sixteen days of storage at 4°C. Asterisks indicate significant differences between treatments according to a One-Way ANOVA statistical test (p < 0.05). [file Image4.jpeg]

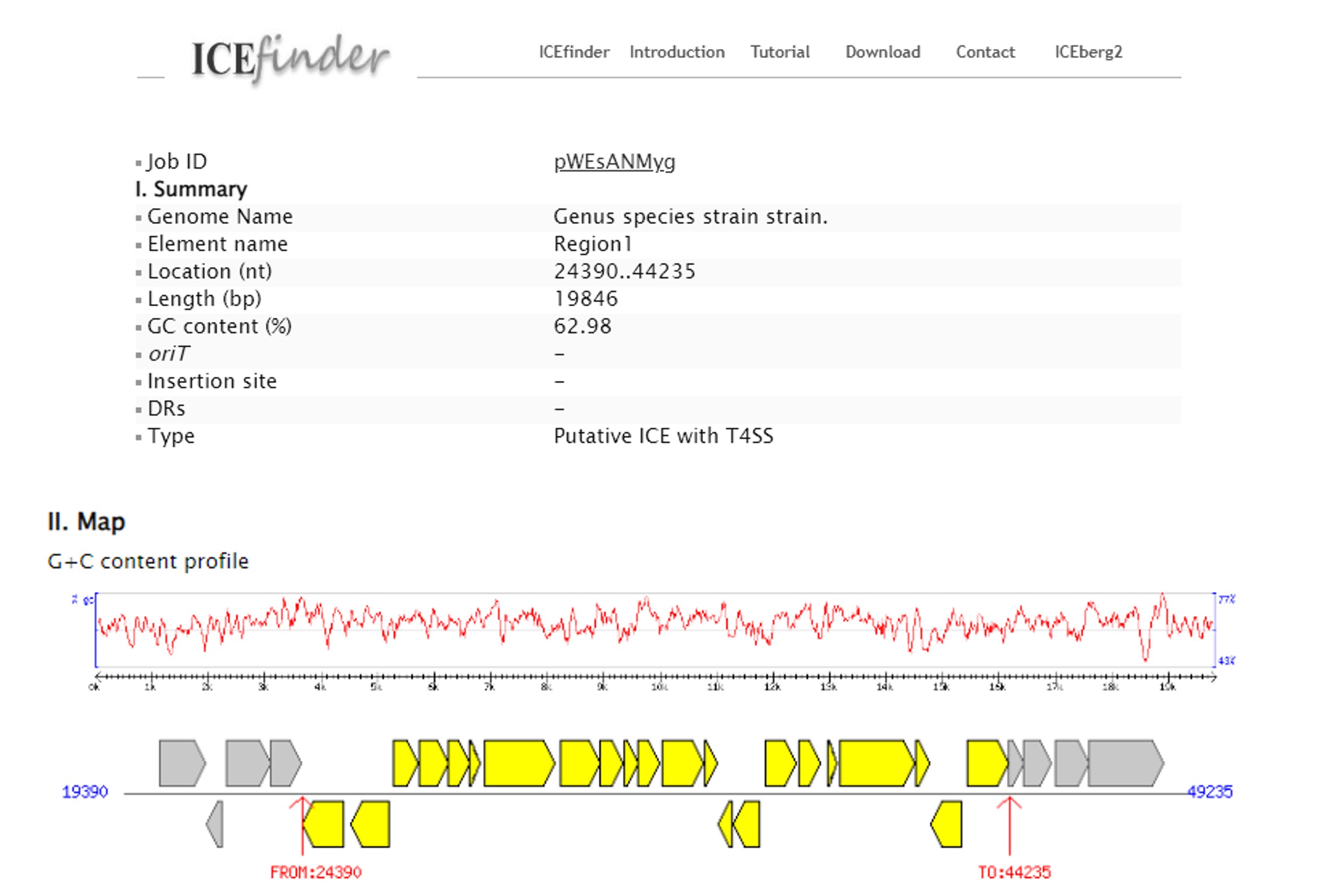

Supplement: Supplementary Figure 5 — Identification of an integrative and conjugative element (ICE) in the genome of ABP-B9 with the ICEfinder tool (https://bioinfo-mml.sjtu.edu.cn/ICEfinder/index.php). [file Image5.jpeg]

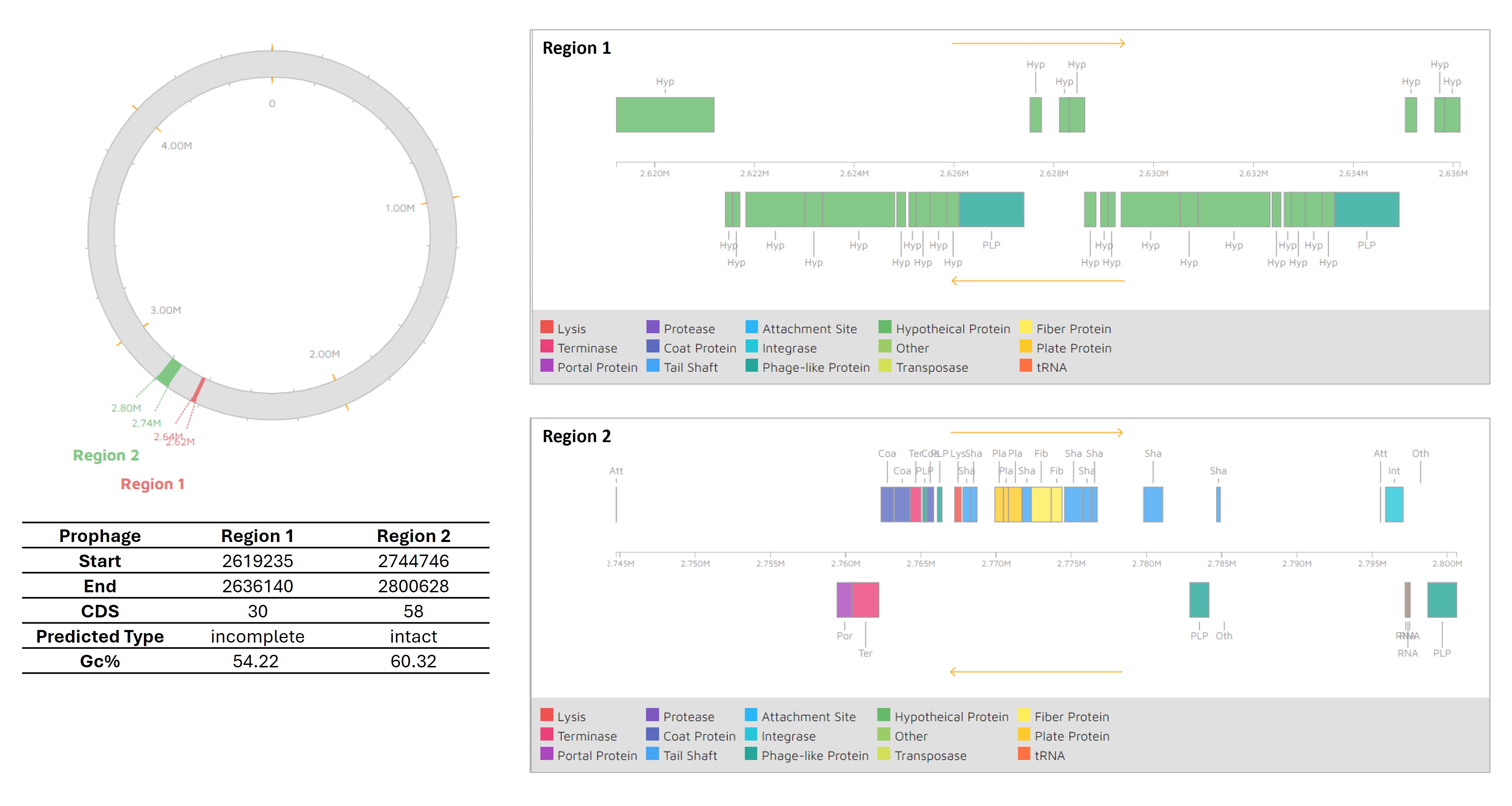

Supplement: Supplementary Figure 6 — Identification of prophage regions in the genome of ABP-B9. Prophago loci were identified with the Phaster tool (https://phaster.ca/). [file Image6.jpeg]

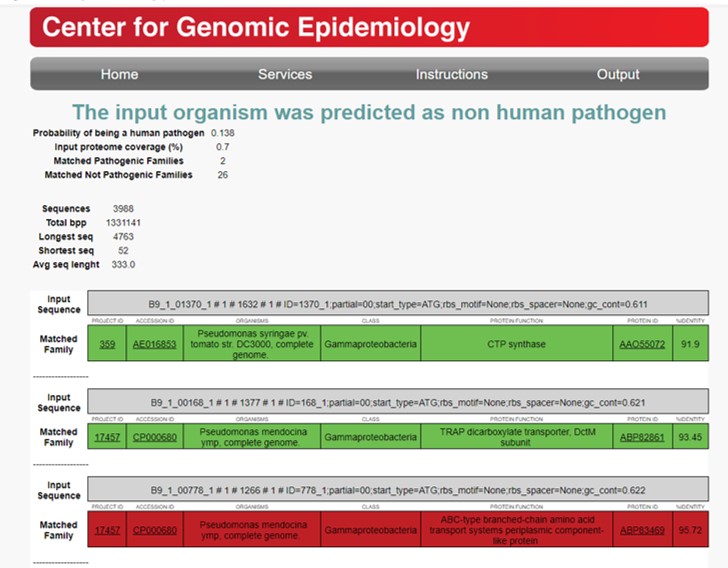

Supplement: Supplementary Figure 7 — Output of PathogenFinder tool from the Center for Genomic Epidemiology (https://cge.food.dtu.dk/services/PathogenFinder/) for the analysis of the ABP-B9 genome. [file Image7.jpeg]

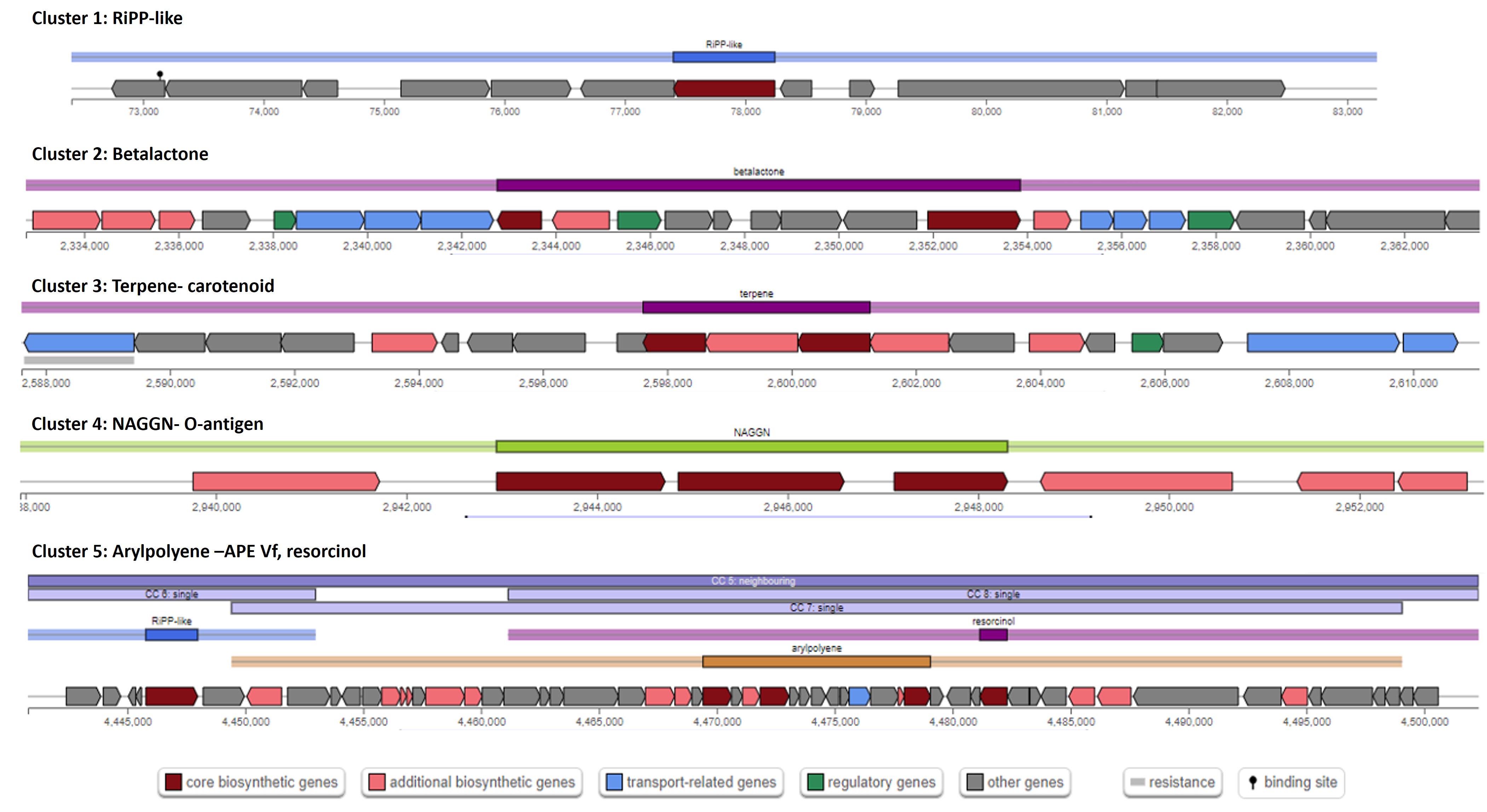

Supplement: Supplementary Figure 8 — Representation of each secondary metabolite biosynthetic gene cluster in the genome of ABP-B9, predicted by antiSMASH. RiPP-like, Ribosomally synthesized and post-translationally modified Peptide; Betalactone; Terpene- carotenoid; NAGGN, N-acetylglutaminylglutamine amide dipeptide; Arylpolyene-APE Vf; resorcinol. [file Image8.jpeg]
