# Supplementary material for: ABP-B9, a new strain of Pseudomonas seleniipraecipitans with biostimulant activity
Source: Front Plant Sci. 2025 Jun 25;16:1561298. doi: 10.3389/fpls.2025.1561298 (PMC12239753; doi:10.3389/fpls.2025.1561298)
Supplement: Supplementary file 9 [file Table1.pdf]

**Supplementary Table 1.** Primers sequences used in this manuscript.

| Name[a] | Sequence (5´-3´) | Product size (bp) | References |
|---|---|---|---|
| **8F** | AGAGTTTGATCCTGGCTCAG | 800 | Turner et al., 1999 |
| **800R** | CTACCAGGGTATCTAAT | | Leis et al., 2023 |
| **AB-858_F** | TGGGAACAGACCTACGTACA | 97 | This study |
| **AB-859_R** | CAGACGGCTTGAAGTGAATCT | | |
| **VP 383_F (Nadh4)** | AGCGTGCTAATCCCTATGTTCAT | 363 | Navarro et al., 2004 |
| **VP 389_R (Nadh4)** | TCGGTGGTTCCTGTTTGGAA | | |

[a]*F, forward primer; R, reverse primer.*

**Supplementary Table 2.** Bioinformatics resources and tools used in the study.

| Server/Tool | Functionality | Data Type | Access Link |
|---|---|---|---|
| **MetaboAnalyst 6.0** | Metabolomics data analysis | Peak Intensity table | https://www.metaboanalyst.ca/MetaboAnalyst/ModuleView.xhtml |
| **BLAST** | Sequence searching | Nucleotide and protein sequences | https://blast.ncbi.nlm.nih.gov/Blast.cgi |
| **Prokka** | Whole genome annotation | Genome sequence | https://github.com/tseemann/prokka |
| **JSpecies** | Measure the probability if two genomes belonging to the same species or not. | Genome sequence | http://www.imedea.uib.es/jspecies |
| **ANICalculator** | Compare two prokaryotic genome sequences when classifying and identifying bacteria by calculating the ANI value of two prokaryotic genome sequences. | Genome sequence | https://www.ezbiocloud.net/tools/ani |
| **GGDC 3.0** | Calculate genomic distances between pairs of genomes | Genome sequence | http://ggdc.dsmz.de |
| **AAI-profiler** | Helps in the classification and definition of taxonomic groups by comparing proteins between different organisms | Genome sequence | http://ekhidna2.biocenter.helsinki.fi/AAI/) |
| **MEGA7** | Evolutionary and phylogenetic analysis | Genome sequence | |
| **FigTree v1.4.3** | Graphical viewer of phylogenetic trees | Genome sequence | http://tree.bio.ed.ac.uk/software/figtree/ |
| **Clustal X (2.1)** | Multiple sequence alignment" | Genome sequence | http://www.clustal.org/clustal2/ |
| **PHASTER** | Identification and annotation of prophage sequences within bacterial genomes and plasmids | Genome sequence | https://phaster.ca/ |
| **ICEfinder** | Identifies mobile genetic elements in bacterial genomes | Genome sequence | https://bioinfo-mml.sjtu.edu.cn/ICEfinder/index.php |
| **ResFinder** | Identification of acquired antibiotic resistance genes. | Genome sequence | https://www.genomicepidemiology.org/ |
| **VRprofile2** | Detection of antibiotic resistance-associated mobilome in bacterial | Genome sequence | https://tool2-mml.sjtu.edu.cn/VRprofile/ |
| **PathogenFinder** | Prediction of a bacteria's pathogenicity towards human hosts. | Genome sequence | https://www.genomicepidemiology.org/services/ |
| **ToxFinder 1.0.** | Identifies genes involved in mycotoxin synthesis. | Genome sequence | https://www.genomicepidemiology.org/services/ |
| **antiSMASH v6.0** | Search a genome sequence for secondary metabolite biosynthetic gene clusters | Genome sequence | https://antismash.secondarymetabolites.org/#!/start |
| **RAST** | Annotating complete or nearly complete bacterial genomes. | Genome sequence | https://rast.nmpdr.org/ |
| **SecRet6** | Detection T6SS gene clusters in an annotated or unannotated genome sequence | Genome sequence | https://bioinfo-mml.sjtu.edu.cn/SecReT6/t6ss_prediction.php |

**Supplementary Table 3.** Summary of the identity percentage obtained from the analysis of the sequences of the *16S rRNA, gyrB, rpoD, and rpoB* genes individually or concatenated between ABP-B9 and different *Pseudomonas* strains using Clustal Omega (Clustal 2.1).

| Gene | Sequence with the highest percentage of identity with ABP-B9 | Accession | Phylogenetic group | Lineage | % of identity |
|---|---|---|---|---|---|
| ***16S rRNA*** | *P. seleniipraecipitans* strain IS1-14 | MF523618.1 | *P. straminea* | *P. fluorescens* | 100 |
| | *P. seleniipraecipitans* strain CA5 | NR_116646.1 | *P. straminea* | *P. fluorescens* | 99.77 |
| | *P. seleniipraecipitans* strain PA100 | KT710798.1 | *P. straminea* | *P. fluorescens* | 99.7 |
| | *Pseudomonas* sp. PA35 | KT710797.1 | Unclassified | Unclassified | 99.23 |
| | *P. punonensis* strain LMT03 | MT677939.1 | *P. straminea* | *P. fluorescens* | 99.07 |
| | *P. fulva* 12-X | CP002727.1_239522-241045 | *P. putida* | *P. fluorescens* | 99.04 |
| ***gyrB*** | *P. seleniipraecipitans* strain LMG 25475T | HE800485.1 | *P. straminea* | *P. fluorescens* | 98.05 |
| | *P. punonensis* strain LMT03 | JX435105.1 | *P. straminea* | *P. fluorescens* | 91.48 |
| | *P. seleniipraecipitans* strain PA100 | KT710837.1 | *P. straminea* | *P. fluorescens* | 91.17 |
| | *P. flavescens* LMG 18387T | FN554183.1 | *P. aeruginosa* | *P. aeruginosa* | 90.73 |
| ***rpoD*** | *P. seleniipraecipitans* strain LMG 25475T | HE800485.1 | *P. straminea* | *P. fluorescens* | 98.02 |
| | *P. punonensis* strain LMT03 | JX435105.1 | *P. straminea* | *P. fluorescens* | 86.61 |
| | *P. fulva* 12-X | NC_015556 | *P. putida* | *P. fluorescens* | 86.22 |
| ***rpoB*** | *P. seleniipraecipitans* strain LMG 25475T | HE800485.1 | *P. straminea* | *P. fluorescens* | 99.12 |
| | *P. punonensis* strain LMT03 | JX435105.1 | *P. straminea* | *P. fluorescens* | 95.51 |
| | *P. straminea* strain LMG 21615T | FN554758.1 | *P. straminea* | *P. fluorescens* | 93.55 |
| ***16s rRNA_gyrB_rpoD_rpoB*** | *P. seleniipraecipitans* strain CA5 | NR_116646.1 | *P. straminea* | *P. fluorescens* | 98.96 |
| | *P. punonensis* strain LMT03 | JX435105.1 | *P. straminea* | *P. fluorescens* | 93.80 |
| | *P. fulva* 12-X | CP002727.1_239522-241045 | *P. putida* | *P. fluorescens* | 92.55 |
| | *P. straminea* | D84023.1 | *P. straminea* | *P. fluorescens* | 92.55 |

**Supplementary Table 4.** Number of enzymes detected in the ABP-B9 genome, involved in different metabolic pathways, based on data from the Kyoto Encyclopedia of Genes and Genomes (KEGG) database, as determined using the RAST bioinformatics tool.

| KEGG map | Distinct ECs | Isolate ABP-B9 |
|---|---|---|
| Biosynthesis of plant hormones | 131 | 66 (50.4 %) |
| Biosynthesis of terpenoids and steroids | 108 | 31 (28.7 %) |
| Biosynthesis of phenylpropanoids | 105 | 48 (45.7 %) |
| Purine metabolism | 104 | 43 (41.3 %) |
| Biosynthesis of alkaloids derived from shikimate pathway | 101 | 43 (42.6 %) |
| Arginine and proline metabolism | 97 | 39 (40.2 %) |
| Amino sugar and nucleotide sugar metabolism | 94 | 23 (24.5 %) |
| Biosynthesis of alkaloids derived from ornithine, lysine and nicotinic acid | 71 | 36 (50.7 %) |
| Starch and sucrose metabolism | 71 | 16 (22.5 %) |
| Porphyrin and chlorophyll metabolism | 66 | 29 (43.9 %) |
| Fructose and mannose metabolism | 65 | 12 (18.5 %) |
| Cysteine and methionine metabolism | 64 | 18 (28.1 %) |
| Pyruvate metabolism | 64 | 21 (32.8 %) |
| Pyrimidine metabolism | 64 | 26 (40.6 %) |
| Tyrosine metabolism | 63 | 13 (20.6 %) |
| Biosynthesis of alkaloids derived from terpenoid and polyketide | 62 | 28 (45.2 %) |
| Biosynthesis of alkaloids derived from histidine and purine | 62 | 45 (72.6 %) |
| Glyoxylate and dicarboxylate metabolism | 58 | 12 (20.7 %) |
| Glycine, serine and threonine metabolism | 57 | 25 (43.9 %) |
| Nitrogen metabolism | 57 | 15 (26.3 %) |
| Pentose and glucuronate interconversions | 56 | 5 (8.9 %) |
| Pentose and glucuronate interconversions | 56 | 5 (8.9 %) |
| Isoquinoline alkaloid biosynthesis | 55 | 5 (9.1 %) |
| Lysine degradation | 54 | 9 (16.7 %) |
| Butanoate metabolism | 52 | 15 (28.8 %) |
| Phenylalanine metabolism | 51 | 6 (11.8 %) |
| Benzoate degradation via hydroxylation | 50 | 4 (8.0 %) |
| Glycerophospholipid metabolism | 50 | 16 (32.0 %) |
| Nicotinate and nicotinamide metabolism | 47 | 14 (29.8 %) |
| Propanoate metabolism | 47 | 16 (34.0 %) |
| Benzoate degradation via CoA ligation | 44 | 8 (18.2 %) |
| Ascorbate and aldarate metabolism | 44 | 10 (22.7 %) |
| Alanine, aspartate and glutamate metabolism | 43 | 22 (51.2 %) |
| Glycolysis / Gluconeogenesis | 41 | 17 (41.5 %) |

| | | |
|---|---|---|
| Glutathione metabolism | 40 | 13 (32.5 %) |
| Inositol phosphate metabolism | 40 | 3 (7.5 %) |
| Histidine metabolism | 37 | 15 (40.5 %) |
| Galactose metabolism | 37 | 7 (18.9 %) |
| Pentose phosphate pathway | 37 | 21 (56.8 %) |
| Glycerolipid metabolism | 36 | 10 (27.8 %) |
| Valine, leucine and isoleucine degradation | 34 | 12 (35.3 %) |
| Methane metabolism | 33 | 7 (21.2 %) |
| beta-Alanine metabolism | 32 | 8 (25.0 %) |
| Aminoacyl-tRNA biosynthesis | 31 | 24 (77.4 %) |
| Lysine biosynthesis | 31 | 12 (38.7 %) |
| Phenylalanine, tyrosine and tryptophan biosynthesis | 31 | 22 (71.0 %) |
| Sulfur metabolism | 30 | 9 (30.0 %) |
| Phenylpropanoid biosynthesis | 30 | 1 (3.3 %) |
| 2,4-Dichlorobenzoate degradation | 29 | 2 (6.9 %) |
| Arachidonic acid metabolism | 29 | 2 (6.9 %) |
| Fatty acid metabolism | 29 | 10 (34.5 %) |
| N-Glycan biosynthesis | 29 | 1 (3.4 %) |
| Sphingolipid metabolism | 29 | 3 (10.3 %) |
| gamma-Hexachlorocyclohexane degradation | 28 | 2 (7.1 %) |
| Ether lipid metabolism | 28 | 1 (3.6 %) |
| Pantothenate and CoA biosynthesis | 28 | 15 (53.6 %) |
| Cyanoamino acid metabolism | 27 | 3 (11.1 %) |
| Terpenoid backbone biosynthesis | 27 | 10 (37.0 %) |
| Vitamin B6 metabolism | 26 | 8 (30.8 %) |
| Flavone and flavonol biosynthesis | 25 | 1 (4.0 %) |
| Folate biosynthesis | 25 | 10 (40.0 %) |
| Ubiquinone and other terpenoid-quinone biosynthesis | 25 | 6 (24.0 %) |
| Carbon fixation in photosynthetic organisms | 25 | 14 (56.0 %) |
| Androgen and estrogen metabolism | 24 | 1 (4.2 %) |
| One carbon pool by folate | 24 | 13 (54.2 %) |
| Flavonoid biosynthesis | 23 | 2 (8.7 %) |
| Phosphatidylinositol signaling system | 23 | 3 (13.0 %) |
| Drug metabolism - other enzymes | 22 | 6 (27.3 %) |
| Limonene and pinene degradation | 22 | 7 (31.8 %) |
| Naphthalene and anthracene degradation | 22 | 3 (13.6 %) |
| Lipopolysaccharide biosynthesis | 22 | 9 (40.9 %) |
| Citrate cycle (TCA cycle) | 22 | 13 (59.1 %) |

| | | |
|---|---|---|
| Caprolactam degradation | 21 | 4 (19.0 %) |
| Fatty acid biosynthesis | 21 | 10 (47.6 %) |
| Selenoamino acid metabolism | 21 | 9 (42.9 %) |
| Styrene degradation | 21 | 3 (14.3 %) |
| Carotenoid biosynthesis | 19 | 4 (21.1 %) |
| Primary bile acid biosynthesis | 19 | 1 (5.3 %) |
| C5-Branched dibasic acid metabolism | 18 | 2 (11.1 %) |
| Peptidoglycan biosynthesis | 18 | 13 (72.2 %) |
| Retinol metabolism | 18 | 2 (11.1 %) |
| Valine, leucine and isoleucine biosynthesis | 18 | 13 (72.2 %) |
| Peptidoglycan biosynthesis | 18 | 13 (72.2 %) |
| 1- and 2-Methylnaphthalene degradation | 17 | 3 (17.6 %) |
| Indole alkaloid biosynthesis | 17 | 1 (5.9 %) |
| Taurine and hypotaurine metabolism | 17 | 4 (23.5 %) |
| alpha-Linolenic acid metabolism | 16 | 4 (25.0 %) |
| Glycosaminoglycan degradation | 16 | 1 (6.2 %) |
| Thiamine metabolism | 16 | 5 (31.2 %) |
| Riboflavin metabolism | 16 | 10 (62.5 %) |
| Biosynthesis of unsaturated fatty acids | 15 | 7 (46.7 %) |
| Tropane, piperidine and pyridine alkaloid biosynthesis | 15 | 1 (6.7 %) |
| Penicillin and cephalosporin biosynthesis | 14 | 2 (14.3 %) |
| Glycosphingolipid biosynthesis - globo series | 14 | 2 (14.3 %) |
| D-Glutamine and D-glutamate metabolism | 13 | 4 (30.8 %) |
| Phosphonate and phosphinate metabolism | 13 | 2 (15.4 %) |
| Reductive carboxylate cycle (CO2 fixation) | 13 | 7 (53.8 %) |
| Atrazine degradation | 12 | 1 (8.3 %) |
| Biotin metabolism | 12 | 9 (75.0 %) |
| Geraniol degradation | 12 | 5 (41.7 %) |
| Oxidative phosphorylation | 12 | 8 (66.7 %) |
| Stilbenoid, diarylheptanoid and gingerol biosynthesis | 12 | 2 (16.7 %) |
| Linoleic acid metabolism | 11 | 1 (9.1 %) |
| Ethylbenzene degradation | 11 | 2 (18.2 %) |
| Glycosphingolipid biosynthesis - ganglio series | 11 | 2 (18.2 %) |
| 1,1,1-Trichloro-2,2-bis(4-chlorophenyl)ethane (DDT) degradation | 10 | 2 (20.0 %) |
| Anthocyanin biosynthesis | 9 | 2 (22.2 %) |
| Bisphenol A degradation | 9 | 2 (22.2 %) |
| Drug metabolism - cytochrome P450 | 9 | 2 (22.2 %) |
| Other glycan degradation | 9 | 1 (11.1 %) |

| | | |
|---|---|---|
| Betalain biosynthesis | 8 | 3 (37.5 %) |
| D-Arginine and D-ornithine metabolism | 8 | 1 (12.5 %) |
| Fatty acid elongation in mitochondria | 8 | 3 (37.5 %) |
| Keratan sulfate biosynthesis | 8 | 1 (12.5 %) |
| O-Glycan biosynthesis | 8 | 1 (12.5 %) |
| Glycosylphosphatidylinositol(GPI)-anchor biosynthesis | 8 | 1 (12.5 %) |
| Polyketide sugar unit biosynthesis | 8 | 5 (62.5 %) |
| Secondary bile acid biosynthesis | 8 | 1 (12.5 %) |
| Biosynthesis of siderophore group nonribosomal peptides | 7 | 2 (28.6 %) |
| Tetrachloroethene degradation | 7 | 1 (14.3 %) |
| Metabolism of xenobiotics by cytochrome P450 | 7 | 2 (28.6 %) |
| Novobiocin biosynthesis | 6 | 5 (83.3 %) |
| D-Alanine metabolism | 6 | 2 (33.3 %) |
| Synthesis and degradation of ketone bodies | 6 | 3 (50.0 %) |
| Insect hormone biosynthesis | 6 | 2 (33.3 %) |
| 1,2-Dichloroethane degradation | 5 | 2 (40.0 %) |
| Benzoxazinoid biosynthesis | 5 | 1 (20.0 %) |
| mTOR signaling pathway | 5 | 1 (20.0 %) |
| Puromycin biosynthesis | 5 | 1 (20.0 %) |
| 3-Chloroacrylic acid degradation | 4 | 2 (50.0 %) |
| High-mannose type N-glycan biosynthesis | 4 | 2 (50.0 %) |
| Lipoic acid metabolism | 4 | 2 (50.0 %) |
| Biosynthesis of type II polyketide backbone | 3 | 1 (33.3 %) |
| Tetracycline biosynthesis | 3 | 1 (33.3 %) |
| Photosynthesis | 3 | 2 (66.7 %) |
| Biosynthesis of ansamycins | 2 | 1 (50.0 %) |
| Biosynthesis of type II polyketide products | 2 | 1 (50.0 %) |
| O-Mannosyl glycan biosynthesis | 2 | 1 (50.0 %) |
| T cell receptor signaling pathway | 2 | 1 (50.0 %) |
| beta-Lactam resistance | 1 | 1 (100.0 %) |
| Biosynthesis of vancomycin group antibiotics | 1 | 1 (100.0 %) |

**Supplementary Table 5.** Genes of ABP-B9 potentially involved in growth promotion and protein secretion

| | Class | Genes | Locus Tag | Product |
|---|---|---|---|---|
| Growth promotion | ACC deamination | dcyD | 02392 | D-cysteine desulfhydrase |
| | Trehalose synthesis | *treS* | 02002 | Trehalose synthase/amylase TreS |
| | | *treY* | 02026 | Maltooligosyl trehalose synthase |
| | | *treZ* | 02028 | Malto-oligosyltrehalose trehalohydrolase |
| | IAA biosynthesis | *trpA* | 00518 | Tryptophan synthase alpha chain |
| | | *trpB* | 00519 | Tryptophan synthase beta chain |
| | | *trpC* | 04015 | Indole-3-glycerol phosphate synthase |
| | | *trpD* | 04016 | Anthranilate phosphoribosyltransferase |
| | | *trpE* | 04032 | Anthranilate synthase component 1 |
| | | *trpF* | 01758 | N-(5'-phosphoribosyl)anthranilate isomerase |
| | | *trpG* | 04017 | Anthranilate synthase component 2 |
| | | *Trpl* | 00528 | HTH-type transcriptional regulator TrpI |
| | | *trpS* | 03830 | Tryptophan--tRNA ligase |
| | Thioredoxin | *ahpF_1* | 02961 | Alkyl hydroperoxide reductase subunit F |
| | | *trxA_1* | 03065 | Thioredoxin |
| | | *resA* | 03274 | Thiol-disulfide oxidoreductase ResA |
| | | *ybbN* | 03925 | putative protein YbbN |
| | | *trxA_2* | 04174 | Thioredoxin 1 |
| | | *trxC* | 04186 | Thioredoxin 2 |
| | | *dsbA* | 00106 | Thiol:disulfide interchange protein DsbA |
| | | *btuE* | 01409 | Thioredoxin/glutathione peroxidase BtuE |
| | | B9_1_01412 | 01412 | Glucosaminate ammonia-lyase |
| | Glutaredoxin | grxC | 0893 | Glutaredoxin 3 |
| | | yffB | 01317 | Protein YffB |
| | | grxD | 01591 | Glutaredoxin 4 |
| | | B9_1_02634 | 02634 | hypothetical protein |
| | | yfgD | 03278 | putative protein YfgD |
| | | B9_1_03534 | 03534 | hypothetical protein |
| | Ammonia assimilation | *gltA_1* | 01820 | Glutamate synthase [NADPH] large chain |
| | | *gltA_2* | 02322 | Citrate synthase |
| | | *gltB* | 04226 | Glutamate synthase [NADPH] large chain |
| | | *gltC_1* | 00693 | HTH-type transcriptional regulator GltC |
| | | *gltC_2* | 03371 | HTH-type transcriptional regulator GltC |
| | | *gltD* | 04225 | Glutamate synthase [NADPH] small chain |
| | | *gltI* | 00224 | Glutamate/aspartate import solute-binding protein |
| | | *gltK* | 00226 | Glutamate/aspartate import permease protein GltK |
| | | *gltP* | 00232 | Proton/glutamate-aspartate symporter |
| | | *gltR_1* | 01071 | HTH-type transcriptional regulator GltR |
| | | *gltR_2* | 01674 | HTH-type transcriptional regulator GltR |
| | | *gltX* | 01737 | Glutamate--tRNA ligase |
| | | *glnA* | 00901 | Glutamine synthetase |
| | | *gdhA* | 01047 | NADP-specific glutamate dehydrogenase |
| | Phosphate solubilization | *pstA_1* | 00581 | Phosphate transport system permease protein PstA |
| | | *pstA_2* | 02969 | Phosphate transport system permease protein PstA |
| | | *pstB* | 00582 | Phosphate import ATP-binding protein PstB |
| | | *pstB3* | 02968 | Phosphate import ATP-binding protein PstB 3 |
| | | *pstC* | 02970 | Phosphate transport system permease protein PstC |

| | | | | |
|---|---|---|---|---|
| | | *pstC1* | 00580 | Phosphate transport system permease protein PstC 1 |
| | | *pstS_1* | 00579 | Phosphate-binding protein PstS |
| | | *pstS_2* | 02971 | Phosphate-binding protein PstS |
| | Urease synthesis | B9_1_01189 | 01189 | Urease |
| | Nitric oxide synthesis | *nirD* | 00845 | Nitrite reductase (NADH) small subunit |
| | | *norG_1* | 00323 | HTH-type transcriptional regulator NorG |
| | | *norG_2* | 02106 | HTH-type transcriptional regulator NorG |
| | | *norG_3* | 03814 | HTH-type transcriptional regulator NorG |
| | | *norG_4* | 03896 | HTH-type transcriptional regulator NorG |
| | | *norR2* | 00440 | Nitric oxide reductase transcription regulator NorR2 |
| | GABA synthesis | *gabP* | 01008 | GABA permease |
| | | *gabR_1* | 01633 | HTH-type transcriptional regulatory protein GabR |
| | | *gabR_2* | 02011 | HTH-type transcriptional regulatory protein GabR |
| | Acetoin synthesis | *acuC* | 03763 | Acetoin utilization protein AcuC |
| | | *ydaP* | 01668 | Putative thiamine pyrophosphate-containing protein YdaP |
| | | *ilvH* | 03744 | Acetolactate synthase isozyme 3 small subunits |
| | | *ilvI* | 03745 | Acetolactate synthase isozyme 3 large subunits |
| Protein secretion systems | Type IV Pilus | *pilA* | 00993 | Fimbrial protein |
| | | *pilE1* | 00953 | Fimbrial protein |
| | | *pilQ* | 00990 | Type IV pilus biogenesis and competence protein PilQ |
| | | *pilT_1* | 00022 | Twitching mobility protein |
| | | *pilT_2* | 00023 | Twitching mobility protein |
| | | *pilY_1_2* | 00954 | Type IV pilus biogenesis factor PilY1 |
| | | B9_1_00391 | 00391 | twitching motility protein |
| | | B9_1_00392 | 00392 | Type IV pilus biogenesis protein PilE |
| | | B9_1_00394 | 00394 | Pilus assembly protein tip-associated adhesin |
| | | B9_1_00401 | 00401 | pilus assembly protein PilX |
| | | B9_1_00955 | 00955 | Fimbrial protein |
| | | B9_1_00956 | 00956 | Major pilin protein fimA |
| | | B9_1_00957 | 00957 | Flp Fap pilin component |
| | | B9_1_03423 | 03423 | Peptidase A24A, prepilin type IV |
| | | B9_1_03423 | 03423 | Cleaves type-4 fimbrial leader sequence and methylates the N-terminal (generally Phe) residue (By similarity) |
| | Sec secretory pathway | *secA_1* | 01220 | Protein translocase subunit SecA |
| | | *secA_2* | 03006 | Protein translocase subunit SecA |
| | | *secB* | 00894 | Protein-export protein SecB |
| | | *secD* | 01476 | Protein translocase subunit SecD |
| | | *secE* | 03978 | Protein translocase subunit SecE |
| | | *secF* | 01477 | Protein translocase subunit SecF |
| | | *secG* | 01187 | Protein-export membrane protein SecG |
| | | *secY* | 03945 | Protein translocase subunit SecY |
| | | *yajC* | 01475 | Sec translocon accessory complex subunit YajC |
| | | *ftsY* | 00565 | Signal recognition particle receptor FtsY |
| | | *ffh* | 03566 | Signal recognition particle protein |
| | | *lepB* | 01567 | Signal peptidase I |
| | | *lspA* | 03857 | Lipoprotein signal peptidase |
| | Twin arginine targeting secretory pathway | *tatA* | 00622 | Sec-independent protein translocase protein TatA |
| | | *tatB* | 00621 | Sec-independent protein translocase protein TatB |
| | | *tatC* | 00620 | Sec-independent protein translocase protein TatC |
| | Large conductance mechanosensitive ion channel | *mscL* | 04074 | Large-conductance mechanosensitive channel |
| | | *tolA* | 03258 | Protein TolA |

| | | | | |
|---|---|---|---|---|
| | Type I secretory system | *tolB* | 03257 | Protein TolB |
| | | *TolC* | 00681 | Outer membrane protein TolC |
| | | *tolQ_1* | 01934 | Protein TolQ |
| | | *tolQ_2* | 03260 | Protein TolQ |
| | | *prsE* | 01542 | Type I secretion system membrane fusion protein PrsE |
| | Type II secretory system | hxcR | 04147 | putative type II secretion system protein HxcR |

**Supplementary Table 6.** Biosynthetic Gene Cluster (BGC) annotated in the ABP-B9 genome with the antiSMASH v7.1.0 tool, in 'relaxed and strict' detection strictness.

| Clusters | Type | Most similar known cluster with MIBiG | Gene location predicted by antiSMASH | Similarity (%) |
|---|---|---|---|---|
| **1** | RiPP-like | N.D. | 72404-83246 | |
| **2** | Betalactone | | 2332764-2363858 | |
| **3** | Terpene | Carotenoid | 2587613-2611264 | 100 |
| **4** | NAGGN | O-antigen | 2937945-2953308 | 21 |
| **5** | RiPP-like<br>Arylpolyene<br>Resorcinol | APE Vf | 4440784-4502310 | 40 |

RiPP: Ribosomally synthesized and Post-translationally modified Peptide. NAGGN: N-acetylglutaminylglutamine amide dipeptide. N.D. no defined known clusters are available.

**Supplementary Table 7.** Genes potentially involved in metal resistance of ABP-B9.

| Metal | Gene | Locus Tag | Product |
|---|---|---|---|
| **Nickel** | *cnrA* | 00072 | Nickel and cobalt resistance protein CnrA |
| | *nikA* | 02382 | Nickel-binding periplasmic protein |
| | *nikC* | 02380 | Nickel transport system permease protein NikC |
| | *rcnA* | 01508 | Nickel/cobalt efflux system RcnA |
| **Copper** | B9_1_ | 00059 | hypothetical protein |
| | B9_1_ | 00060 | hypothetical protein |
| | *petE* | 00061 | Plastocyanin |
| | *actP_1* | 00063 | Copper-transporting P-type ATPase |
| | *actP_2* | 00068 | Copper-transporting P-type ATPase |
| | *csoR* | 00073 | Copper-sensing transcriptional repressor CsoR |
| | *copA_1* | 00080 | Copper resistance protein A |
| | *copB* | 00082 | Copper resistance protein B |
| | *yfiH* | 00966 | Polyphenol oxidase |
| | *copA_2* | 01951 | Copper-exporting P-type ATPase |
| | B9_1_ | 02649 | Pseudoazurin |
| | *mco* | 03350 | Multicopper oxidase mco |
| | *copZ* | 03757 | Copper chaperone CopZ |
| | *copA_3* | 03759 | Copper-exporting P-type ATPase |
| | B9_1_ | 03835 | Azurin |
| **Cadmium** | *rcnA* | 00072 | Nickel/cobalt efflux system RcnA |
| | *cnrA* | 01508 | Nickel and cobalt resistance protein CnrA |
| | *nikC* | 02380 | Nickel transport system permease protein NikC |
| | *nikA* | 02382 | Nickel-binding periplasmic protein |
| **Zinc** | *prtS* | 02678 | Protease PrtS |
| | *ftsH_1* | 00066 | ATP-dependent zinc metalloprotease FtsH |
| | B9_1_ | 00110 | hypothetical protein |
| | *znuA* | 00174 | High-affinity zinc uptake system protein ZnuA |
| | *zur* | 00175 | Zinc uptake regulation protein |
| | *znuC_1* | 00176 | Zinc import ATP-binding protein ZnuC |
| | *znuB_1* | 00177 | High-affinity zinc uptake system membrane protein ZnuB |
| | *yeiR* | 00271 | Zinc-binding GTPase YeiR |
| | B9_1_ | 00563 | putative zinc protease |
| | B9_1_ | 00564 | putative zinc protease |
| | B9_1_ | 00796 | hypothetical protein |
| | *znuC_2* | 02955 | Zinc import ATP-binding protein ZnuC |
| | *znuB_2* | 02956 | High-affinity zinc uptake system membrane protein ZnuB |
| | B9_1_ | 03157 | putative zinc-binding alcohol dehydrogenase |
| | B9_1_ | 03181 | Zinc-type alcohol dehydrogenase-like protein |
| | *ftsH_3* | 04001 | ATP-dependent zinc metalloprotease FtsH |
| **Molybdate** | *modB_1* | 00479 | Molybdenum transport system permease protein ModB |
| | *modA* | 00480 | Molybdate-binding protein ModA |
| | *modE* | 00930 | Transcriptional regulator ModE |
| | *modB_2* | 03351 | Molybdenum transport system permease protein ModB |
| **Cobalt** | *rcnA* | 00072 | Nickel/cobalt efflux system RcnA |
| | *cbiG* | 00405 | Cobalt-precorrin-5A hydrolase |
| | B9_1_ | 00406 | hypothetical protein |

| | | | |
|---|---|---|---|
| | B9_1_ | 00407 | hypothetical protein |
| | *cobN* | 00410 | Aerobic cobaltochelatase subunit CobN |
| | *cbiO* | 00775 | Cobalt import ATP-binding protein CbiO |
| | *corC* | 01328 | Magnesium and cobalt efflux protein CorC |
| | *cnrA* | 01508 | Nickel and cobalt resistance protein CnrA |
| | *corA* | 02138 | Cobalt/magnesium transport protein CorA |
| | *zntB* | 02287 | Zinc transport protein ZntB |
| **Arsenate** | *arsA* | 00253 | Arsenical pump-driving ATPase |
| | *arsC* | 00852 | Arsenate reductase |
| | *arsB* | 00853 | Arsenical pump membrane protein |
| | *arsR2* | 00854 | Arsenic resistance transcriptional regulator ArsR2 |
| | *yffB* | 01317 | Protein YffB |
| | *yfgD* | 03278 | putative protein YfgD |
| **Chromate** | *chrA* | 03523 | Chromate transport protein |
